# An RNA-targeting ADP-ribosyltransferase toxin uncouples mRNA stability from productive translation

**DOI:** 10.1093/nar/gkag1177

**Published:** 2026-11-14

**Authors:** Fanyang Lv, Guchen Weng, John C Whitney

**Affiliations:** Michael DeGroote Institute for Infectious Disease Research, McMaster University, Hamilton, ON L8S 4K1, Canada; Department of Biochemistry and Biomedical Sciences, McMaster University, Hamilton, ON L8S 4K1, Canada; Department of Biochemistry and Biomedical Sciences, McMaster University, Hamilton, ON L8S 4K1, Canada; Michael DeGroote Institute for Infectious Disease Research, McMaster University, Hamilton, ON L8S 4K1, Canada; Department of Biochemistry and Biomedical Sciences, McMaster University, Hamilton, ON L8S 4K1, Canada; David Braley Centre for Antibiotic Discovery, McMaster University, Hamilton, ON L8S 4K1, Canada

## Abstract

Messenger RNA (mRNA) modifications regulate transcript stability and translation efficiency across diverse biological systems, but the consequences of toxin-mediated mRNA modification in bacteria remain poorly understood. RhsP2 was recently identified as an RNA-targeting interbacterial ADP-ribosyltransferase toxin, but whether mRNAs are physiological substrates remained unknown. Here, we show that RhsP2 targets mRNA coding sequences and in doing so prevents their efficient translation. eCLIP-seq reveals preferential association of RhsP2 with UAAG-enriched motifs, and biochemical analyses showed that these sequences are preferred substrates for ribose 2′-OH ADP-ribosylation. Unexpectedly, modified mRNAs were broadly stabilized despite impaired protein synthesis, demonstrating that RhsP2 uncouples transcript stability from productive translation. Monosome- and disome-seq analyses show that RhsP2-modified mRNAs induce ribosome stalling and collision, and *in vitro* translation reactions demonstrate that ADP-ribosylation of mRNAs is sufficient to block translation elongation. Together, these findings establish mRNA ADP-ribosylation as a mechanism of toxin-mediated translational sabotage in which chemically modified transcripts accumulate while disrupting protein synthesis.

## Graphical Abstract

**Graphical Abstract:**
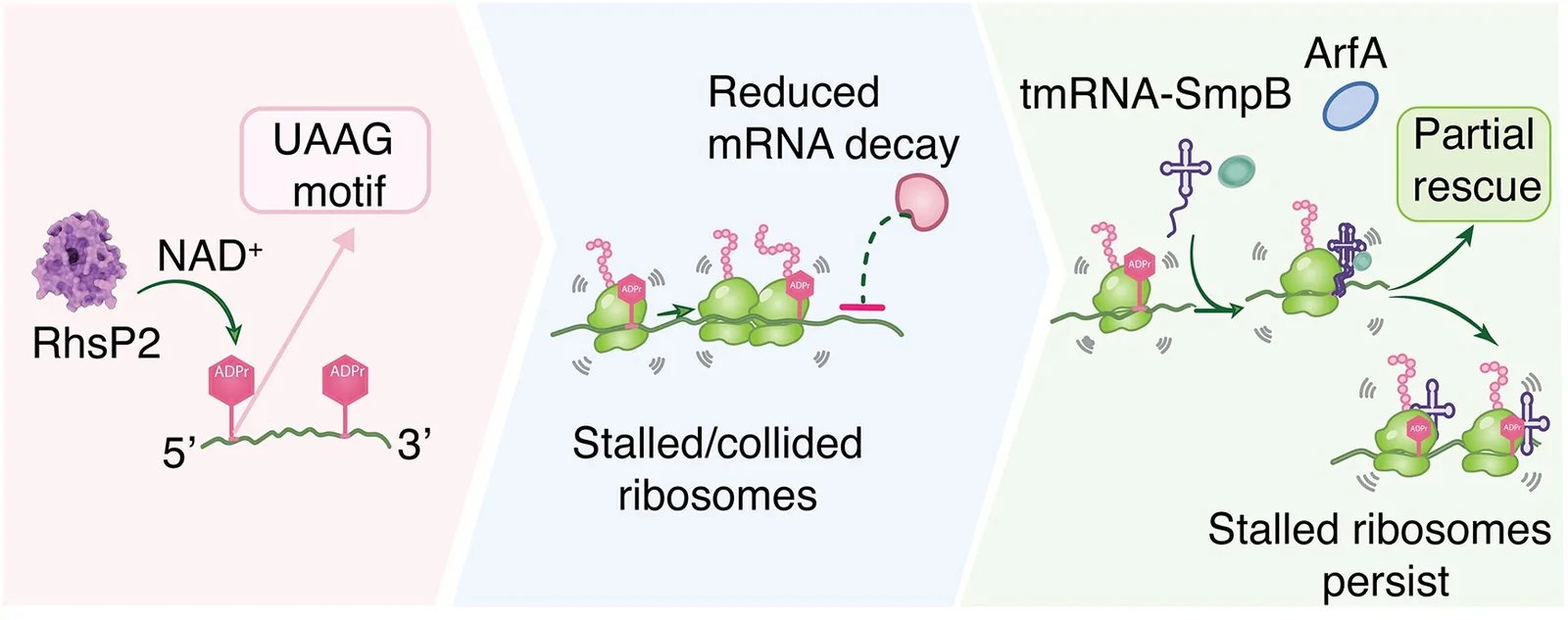

## Introduction

RNA modifications regulate RNA stability and function across all domains of life [1–6]. In bacteria, studies of RNA modification have focused primarily on those found in abundant structured RNAs [7–10] and specialized 5′ RNA caps that influence transcript stability and decay [11–15]. By contrast, the consequences of covalent modification within messenger RNA (mRNA) coding regions remain poorly understood. Because bacterial transcription and translation are tightly coupled [16], chemical alteration of coding region mRNAs has the potential to simultaneously perturb transcript stability, translation elongation, and ribosome dynamics [17, 18].

Many bacterially produced antibacterial toxins inhibit translation by targeting cellular RNAs, often through endoribonucleolytic cleavage of mRNAs, transfer RNAs (tRNAs), or ribosomal RNAs (rRNAs) [19]. Canonical toxin–antitoxin RNases such as MazF, RelE, and VapC arrest bacterial growth by cleaving cellular RNAs and disrupting translation [20–24]. By contrast, covalent RNA modification by antibacterial toxins has the potential to disable translation without degrading the transcript, raising the possibility that chemically altered RNAs could persist within the cell while continuing to associate with translating ribosomes. Such modified RNAs could impose a sustained burden on ribosome homeostasis, but whether toxin-mediated RNA modification produces this state remains unknown.

Although ADP-ribosyltransferases are best known as protein-modifying enzymes that transfer ADP-ribose from NAD⁺ onto target molecules, recent work has identified several antibacterial toxins that ADP-ribosylate RNA [25–30]. These include Tre23, an interbacterial Rhs toxin that modifies 23S rRNA within the GTPase-associated center [31], and CmdT, an anti-bacteriophage toxin that modifies adenines within GA dinucleotides of single-stranded mRNAs, thereby blocking translation [32]. Another RNA-targeting ADP-ribosyltransferase, the type VI secretion system-delivered interbacterial toxin RhsP2, catalyzes ribose 2′-OH ADP-ribosylation of structured non-coding RNAs in competitor bacteria [26, 30]. Previous work identified abundant non-coding RNAs, including tRNAs and regulatory RNAs, as RhsP2 substrates, but whether RhsP2 also targets cellular mRNAs is not known. Moreover, whether direct ADP-ribosylation of mRNA coding sequences (CDSs) is sufficient to disrupt translation has not been established.

Coding region RNA modifications are uniquely positioned to influence gene expression because they are encountered directly by elongating ribosomes. Chemically damaged or modified nucleotides within mRNAs may impair decoding or translocation, thereby promoting ribosome stalling and collision [18]. In bacteria, prolonged elongation stalls can further trigger ribosome rescue pathways, including tmRNA-mediated *trans*-translation and alternative rescue systems such as ArfA [33, 34]. However, it remains unclear whether toxin-catalyzed modification of mRNA CDSs primarily alters transcript stability, ribosome dynamics, or both.

In this study, we show that the antibacterial toxin RhsP2 targets bacterial mRNAs by preferentially associating with UAA-enriched coding regions and modifying these transcripts. Unexpectedly, rather than promoting mRNA degradation, RhsP2-mediated modification stabilizes target transcripts despite reduced protein synthesis. Ribosome profiling and *in vitro* reconstitution of translation demonstrate that ADP-ribosylation of mRNAs impedes their elongation, resulting in ribosome stalling, collision, and induction of ribosome rescue pathways. Together, these findings reveal that toxin-mediated mRNA modification can disrupt gene expression without eliminating the targeted transcript.

## Materials and methods

### Bacterial strains and growth conditions

Cultures were grown in lysogeny broth (LB) medium containing 10 g/l tryptone, 10 g/l NaCl, and 5 g/l yeast extract at 37°C. Solid LB medium contained 1.5% (w/v) agar. *Escherichia coli* XL1-Blue was used for plasmid maintenance and toxicity assays. *Escherichia coli* BL21 (DE3) pLysS was used for protein expression and purification. All *E. coli* strains were grown in LB broth with shaking at 220 rpm or on LB agar plates at 37°C or 18°C, as required. Where required, media were supplemented with antibiotics or inducers at the following final concentrations: carbenicillin, 150 µg/ml; trimethoprim, 200 µg/ml; gentamicin, 30 µg/ml; ampicillin, 100 µg/ml; chloramphenicol, 15 µg/ml; isopropyl β-D-1-thiogalactopyranoside (IPTG), 1 mM; L-rhamnose, 0.2% (w/v); or L-arabinose, 0.2% (w/v). A detailed list of strains used in this study is provided in Supplementary Table S1.

### DNA manipulation, plasmid construction, and mutant strain generation

All primers were synthesized by Integrated DNA Technologies (IDT). DNA polymerase and restriction enzymes were obtained from New England Biolabs (NEB). Sanger sequencing was performed by The Centre for Applied Genomics. Plasmids used for heterologous expression included pETDuet-1, pSCrhaB2-CV, pPSV39-CV, and pBAD33. Plasmids were constructed using Gibson Assembly or NEBuilder HiFi DNA Assembly, as indicated. Correct plasmid assembly and sequence integrity were confirmed by Sanger sequencing. A detailed list of plasmids used in this study is provided in Supplementary Table S2.

### Growth curve assays

Growth curve assays were performed to assess the toxicity of RhsP2 and the indicated variants. Overnight cultures of *E. coli* strains harboring plasmids encoding RhsP2, RhsP2 variants, or the corresponding empty-vector control were diluted into fresh LB medium supplemented with the appropriate antibiotics and grown at 37°C with shaking. Cell growth was monitored by manually measuring OD_600_ at 30-min or 1-h intervals. After 4 h of growth, expression of RhsP2 or its variants was induced by addition of L-rhamnose to a final concentration of 0.2% (w/v). Cultures were then continuously incubated at 37°C with shaking, and OD_600_ measurements were continued at the indicated time intervals. Growth curves were generated by plotting OD_600_ values over time.

### Protein expression and purification

For purification of wild-type (WT) RhsP2_tox_ and its point mutants, each toxin variant was co-expressed with its cognate RhsP2 immunity protein from pETDuet to prevent cellular intoxication. An on-column denaturation and refolding strategy was used to remove the immunity protein and purify the N-terminally His-tagged effector. For each toxin-immunity pair, 1 l of LB medium containing 100 µg/ml ampicillin was inoculated with *E. coli* BL21 pLysS cells harboring the toxin-immunity pETDuet construct. Cultures were grown at 37°C with shaking until reaching an OD_600_ of 0.5–0.8. The temperature was then reduced to 18°C, and protein expression was induced by addition of IPTG to a final concentration of 1 mM. After overnight incubation, cells were harvested by centrifugation at 4500 rpm for 15 min and resuspended in lysis buffer (50 mM Tris–HCl, pH8.0, 300 mM NaCl, 10 mM imidazole). Cells were lysed by sonication and the lysates were clarified by centrifugation at 15 000 rpm for 45 min. The supernatant was loaded onto a 1.5 ml gravity flow Ni-NTA agarose column pre-equilibrated with lysis buffer. The column-bound toxin-immunity complex was denatured with 8 M urea supplemented in the lysis buffer, facilitating removal of the untagged immunity protein. Following denaturation, the effector was renatured by washing the column three times with lysis buffer. Refolded protein was eluted using 10 ml of lysis buffer supplemented with 400 mM imidazole. Protein purity was assessed by sodium dodecyl sulfate-polyacrylamide gel electrophoresis (SDS–PAGE) followed by Coomassie Brilliant Blue staining. Imidazole was removed by dialysis into storage buffer containing 20 mM Tris–HCl, pH 7.5, and 150 mM NaCl.

### Electrophoretic mobility shift assay

McaS RNA was transcribed *in vitro* using the TranscriptAid T7 High Yield Transcription kit (Thermo Scientific) according to the manufacturer’s instructions and then purified using a Zymo RNA Clean & Concentrator-5. For electrophoretic mobility shift assays (EMSAs), 0.2 µM McaS RNA was incubated with the indicated concentrations of WT or mutant RhsP2_tox_ protein for 20 min at room temperature in a 10-µl reaction containing 50 mM Tris–HCl, pH 7.5, 50 mM NaCl, 1 mM dithiothreitol (DTT), 5 mM MgCl_2_, and 4% glycerol. The RNA–protein complexes were separated by 6% native PAGE gel at 200 V for 30 min in 0.5× Tris-borate-EDTA (TBE) buffer. Gels were stained with SYBR Gold and imaged using a ChemiDoc MP Imaging System (Bio-Rad). Each experiment was performed with at least two independent biological replicates (*n* ≥ 2). Apparent dissociation constants (*K*_d_) were determined by band densitometry using ImageJ. Bands representing protein-bound or free RNA were quantified, and background-subtracted signals were used to calculate the fraction bound using the following equation: bound/ (bound + unbound). These values were plotted against the concentration of WT or mutant RhsP2tox and fit by nonlinear regression in Prism 9.0 to obtain the *K*_d_ value and *B*_max_ (the fraction bound at which the data plateaus). The fitting equation used was: ${\mathrm{Fraction\ bound\ }}( {\mathrm{\% }} ) = ( {\frac{{{{{\mathrm{B}}}_{{\mathrm{max}}}}[ {{\mathrm{RhsP}}{{2}_{{\mathrm{tox}}}}} ]}}{{{{{\mathrm{K}}}_{\mathrm{d}}} + [ {{\mathrm{RhsP}}{{2}_{{\mathrm{tox}}}}} ]}}} )100.$

For motif-dependent RNA-binding assays shown in Fig. 1F, synthetic 25-nt RNA substrates containing the UAAG-centered sequence or the indicated sequence variants were incubated with increasing concentrations of catalytically inactive RhsP2* (0–10 µM) under the same binding conditions described earlier.

**Figure 1.**
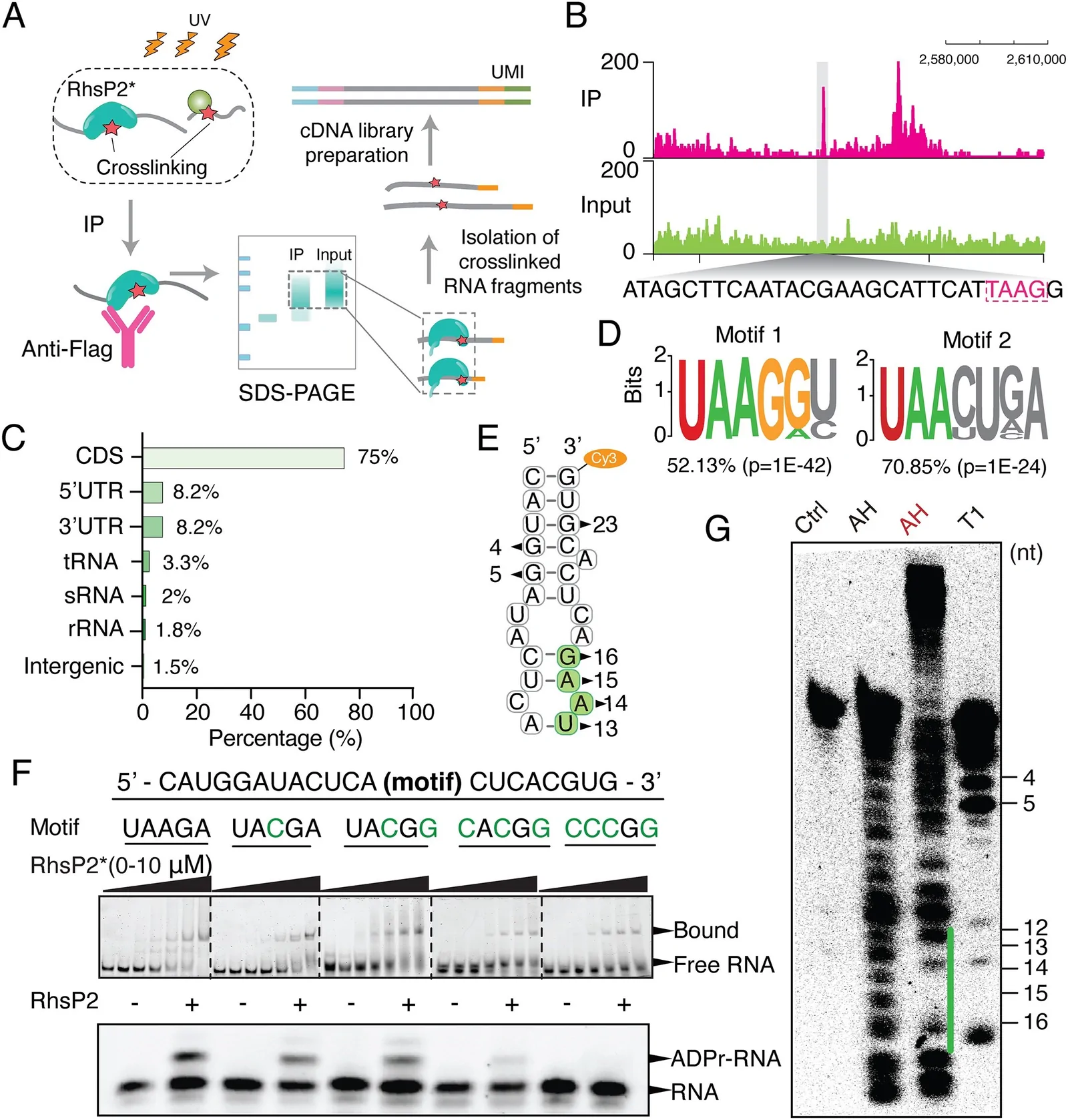
Transcriptome-wide mapping and biochemical characterization of RhsP2 RNA recognition and ADP-ribosylation. (**A**) Schematic of the eCLIP-seq workflow used to map RhsP2-associated RNAs. Cells expressing FLAG-tagged, catalytically inactive RhsP2* were UV-crosslinked, lysed, and subjected to anti-FLAG immunoprecipitation. Crosslinked RNA fragments associated with RhsP2* were recovered following SDS–PAGE separation, converted into complementary DNA (cDNA) libraries containing unique molecular identifiers, and sequenced. (**B**) Representative genome-browser view of RhsP2* eCLIP-seq signal at a target locus. IP and matched input tracks are shown. The shaded region highlights a representative RhsP2*-enriched peak, with the local sequence shown below. (**C**) Genomic annotation of RhsP2* eCLIP-seq peaks across transcript features. The bar plot shows the fraction of called peaks assigned to CDSs, 5′ UTRs, 3′ UTRs, tRNAs, sRNAs, rRNAs, and intergenic regions. (**D**) *De novo* motif analysis of RhsP2* eCLIP-seq peaks. Two enriched UAA-centered motifs identified from RhsP2* eCLIP-seq peaks are shown, including a highly enriched UAAG-containing motif. The percentage of peaks containing each motif and the associated enrichment *P*-value are shown. (**E**) Schematic of the Cy3-labeled synthetic RNA substrate used for biochemical validation and site-mapping experiments. The UAAG motif is highlighted in green, and nucleotide positions used for RNase T1 and alkaline hydrolysis (AH) mapping are indicated. (**F**) Biochemical evaluation of motif-dependent RNA recognition and modification. Top, EMSA evaluating the binding of catalytically inactive RhsP2* to RNA substrates containing the indicated motif variants. RNA substrates were incubated with increasing concentrations of purified RhsP2* (0–10 µM). Free RNA and protein-bound RNA species are indicated. Bottom, *in vitro* ADP-ribosylation of the corresponding RNA substrates. Reactions were performed with 1 mM NAD⁺ in the absence or presence of 0.1 µM purified WT RhsP2 for 10 s, and reaction products were resolved by denaturing urea-PAGE. Unmodified RNA and slower-migrating ADP-ribosylated RNA (ADPr-RNA) species are indicated. (**G**) AH and RNase T1 mapping of purified ADPr-RNA. Unmodified control RNA (Ctrl) and ADPr-RNA were subjected to AH, and RNase T1 digestion was used as a nucleotide reference ladder. The red AH label denotes the AH lane generated from ADPr-RNA. The green bar marks the UAAG-containing region that is protected from alkaline cleavage following ADP-ribosylation. Numbers indicate nucleotide positions within the synthetic RNA substrate shown in panel (E).

### *In vitro* ADP-ribosylation assay

Short 25-nt RNA substrates and related mutant variants were synthesized by IDT. For fluorescent labeling, 50 pmol of the RNA was heated at 90°C for 2 min and then ligated to 100 pmol pCp-Cy3 (Jena Biosciences) using 68 U T4 RNA Ligase 1, single-stranded RNA (ssRNA) Ligase (High Concentration) (NEB). Ligation reactions were performed overnight at 16°C in a 1× buffer containing 50 mM Tris–HCl, pH 7.5, 10 mM MgCl_2_, 2 mM DTT, 2 mM ATP, and 10% dimethyl sulfoxide (DMSO). Labeled RNA was purified by denaturing urea-PAGE. Briefly, ligation products were resolved on a 10% urea-PAGE gel, and Cy3-positive bands were excised. Gel slices were crushed and soaked in 1× RNA elution buffer containing 200 mM NaCl, 10 mM Tris–HCl, pH 7.5, and 1 mM ethylenediaminetetraacetic acid (EDTA), pH 8.0 with shaking at room temperature for 20 min. Eluted RNA was recovered by ethanol precipitation, and the RNA pellet was resuspended in nuclease-free water.

*In vitro* ADP-ribosylation reactions were performed using Cy3-labeled RNA substrates. Before each reaction, RNA was refolded by heating at 90°C for 2 min followed by snap cooling on ice for at least 5 min. Unless otherwise indicated, reactions were assembled in a final volume of 10 µl in ADP-ribosylation buffer containing 50 mM Tris–HCl, pH 7.5, 50 mM NaCl, 1 mM DTT, and 5 mM MgCl_2_. Standard reactions contained 0.1 µM purified RhsP2 protein, 1 pmol Cy3-labeled RNA substrate, and 1 mM NAD^+^. Reactions were incubated at room temperature for 20 min and stopped by heating at 95°C for 5 min.

For kinetic analysis, ADP-ribosylation reactions were performed in duplicate at room temperature, under the indicated RhsP2 and RNA concentrations. RNA substrates were refolded by heating at 90°C for 2 min and cooling to room temperature for 5 min before initiating the reaction. Reactions were initiated by addition of NAD^+^ to a master reaction mixture containing Cy3-labeled RNA substrate and purified RhsP2 in 1× ADP-ribosylation buffer. At each time point, the reactions were quenched on ice with an equal volume of 2× quench buffer containing 10% sucrose, 0.5× TBE, 30 mM EDTA, 8 M urea, 0.14 mg/ml bromophenol blue, and 0.14 mg/ml xylene cyanol. Reaction products were resolved by denaturing 10% urea-PAGE containing 8 M urea. Cy3-labeled unmodified and ADPr-RNA bands were quantified using ImageJ. The fraction of modified RNA was calculated as the intensity of the ADP-ribosylated product divided by the total RNA signal, product plus unmodified substrate. Data were fit to a one-phase exponential association model, *Y* = *Y*_max_ (1 − e^−kobs·t^), using GraphPad Prism 9 to determine the observed first-order rate constant, kobs.

### Alkaline hydrolysis and RNase T1 mapping of ADP-ribosylated RNA

Cy3-labeled 25-nt RNA substrates were generated by pCp-Cy3 ligation and gel purification as described earlier. Purified Cy3-labeled RNA was subjected to *in vitro* ADP-ribosylation using purified RhsP2 in ADP-ribosylation buffer containing 50 mM Tris–HCl, pH 7.5, 50 mM NaCl, 1 mM DTT, and 5 mM MgCl_2_, supplemented with NAD⁺. After incubation for 20 min, ADPr-RNA products were purified using the RNA Clean & Concentrator-5 kit according to the manufacturer’s instructions.

Purified unmodified and ADP-ribosylated Cy3-labeled RNA samples were used for AH and RNase T1 mapping. For AH, RNA samples were incubated with 50 mM NaOH at 90°C for 2 min. Reactions were immediately quenched by adding an equal volume of 2× colorless denaturing gel-loading solution containing 8 M urea and 20 mM EDTA, pH 8.0, and were promptly transferred to −20°C until gel loading.

RNase T1 mapping was performed by incubating 1 pmol labeled RNA with 1 unit of RNase T1 in 1× sodium citrate buffer containing 25 mM sodium citrate pH 5.0 at 55°C for 5 min. Reactions were quenched with 2× colorless denaturing gel-loading solution. Reaction products were resolved on denaturing 15% urea-PAGE gels and imaged using a ChemiDoc MP Imaging System. Site-specific ADP-ribosylation was inferred by comparing the AH cleavage patterns of ADPr-RNA and unmodified control RNA relative to the RNase T1 sequence ladder.

### Reporter construct cloning and preparation of ADP-ribosylated mRNA templates

Dual-tagged reporter constructs were designed to contain a T7 promoter, ribosome-binding site, N-terminal V5 epitope tag, mClover, a variable insert region, and a C-terminal 3× FLAG tag. The variable insert was positioned between mClover and 3× FLAG to distinguish N-terminal translation products from full-length proteins that had translated through the insert region. Reporter inserts encoding a stop control, a non-stalling control sequence, six GUAAGA repeats, or a SecM-derived stalling sequence were assembled individually using NEBuilder® HiFi DNA Assembly Master Mix (NEB) into a pBAD33 plasmid digested with XbaI and HindIII. Correct assembly and reading frame of each reporter construct were confirmed by sequencing. After sequence verification, the corresponding plasmids were used as templates to polymerase chain reaction (PCR)-amplify DNA fragments. PCR products were purified and used for *in vitro* transcription with the TranscriptAid T7 High Yield Transcription kit (Thermo Scientific) according to the manufacturer’s instructions. Following transcription, template DNA was removed by DNase treatment, and mRNAs were purified using Zymo RNA Clean & Concentrator-5, eluted in nuclease-free water, and quantified.

Purified mRNAs were ADP-ribosylated *in vitro* by incubation with recombinant RhsP2 in the presence of NAD⁺. Briefly, equal amounts of mRNA were incubated with 0.1 µM purified RhsP2 and 1mM NAD⁺ in buffer containing 50 mM Tris–HCl, pH 7.5, 50 mM NaCl, 1 mM DTT, and 5 mM MgCl_2_ at room temperature for 20 min. Following the reaction, RNAs were purified again using Zymo RNA Clean & Concentrator-5. The purified RNAs were quantified, and equal amounts of unmodified or modified mRNAs were used as templates for PURExpress® *in vitro* translation reactions.

### Polysome profiling

Cells were grown at 37°C in 50 ml LB medium containing the appropriate antibiotics to an OD_600_ of ~0.45–0.5. Expression of RhsP2 or the corresponding vector control was induced by addition of 0.2% (w/v) L-rhamnose for 20 min. Cells were harvested by centrifugation, flash-frozen in liquid nitrogen, and lysed on ice in polysome lysis buffer containing 20 mM Tris–HCl, pH 8.0, 10 mM MgCl_2_, 100 mM NH_4_Cl, 5 mM CaCl_2_, 100 U/ml DNase I, and 1 mM chloramphenicol. Lysates were clarified by centrifugation at 20 000 × *g* for 30 min at 4°C to remove cell debris.

Sucrose density gradients of 10%–50% were prepared using a Gradient Master 108 (BioComp) in gradient buffer containing 20 mM Tris–HCl, pH 8.0, 10 mM MgCl_2_, 100 mM NH_4_Cl, and 2 mM DTT. Clarified lysate corresponding to 12 AU was loaded onto each gradient and centrifuged in an SW41 rotor at 35 000 rpm for 3 h at 4°C. Gradients were manually fractionated from the top, and absorbance at 254 nm was continuously monitored during fractionation. Fractions were collected across the gradient for downstream analysis. Where indicated, RNA and protein were extracted from individual fractions using TRIzol LS reagent according to the manufacturer’s instructions and analyzed by western blotting or other downstream assays.

### RNA and protein extractions from sucrose fraction

RNA and protein were isolated from sucrose gradient fractions using TRIzol LS reagent. Briefly, 750 μl Trizol LS was added to gradient fractions, vortexed, and incubated at room temperature for 1 min. Chloroform was then added at 150 µl per sample, and samples were vortexed and centrifuged at 14 000 × *g* for 10 min at 4°C to separate the mixture into an upper aqueous phase and a lower organic fraction. After phase separation, the upper aqueous phase was used for RNA isolation, whereas the remaining organic fraction from the same tube was retained for protein recovery.

For RNA isolation, the upper aqueous phase was carefully transferred to a new tube. GlycoBlue and an equal volume of isopropanol were added, and RNA was precipitated by incubation for 30 min on ice. Samples were then centrifuged at 14 000 × *g* for 30 min at 4°C to pellet RNA. RNA pellets were washed twice with 75% ethanol, air-dried briefly, and resuspended in nuclease-free water.

For protein isolation, the remaining organic fraction from the same tube was retained after removal of the aqueous phase. Proteins were recovered from this fraction according to the TRIzol LS reagent protocol. Briefly, proteins were precipitated from the organic-phase-derived fraction, washed to remove residual phenol and salts, and resuspended in SDS loading buffer. Protein samples were then analyzed by SDS–PAGE and immunoblotting.

### PURExpress *in vitro* translation and ribosome complex fractionation

Reporter mRNA templates used for *in vitro* translation were prepared and ADP-ribosylated as described earlier. *In vitro* translation reactions were performed using the PURExpress *In Vitro* Protein Synthesis Kit according to the manufacturer’s instructions. Reactions were assembled in a final volume of 25 µl using purified reporter mRNAs as templates, and each reaction was supplemented with 20 U Murine RNase Inhibitor.

For analysis of total translation products, 5 µg of unmodified or modified mRNA was added to each reaction and incubated at 37°C for 60 min. Reactions were stopped on ice, mixed with SDS loading buffer, boiled at 95°C for 5 min, and analyzed by SDS–PAGE followed by anti-V5 and anti-FLAG immunoblotting.

For ribosome-complex fractionation, reactions were performed similarly except that 10 µg of reporter mRNA was used to increase recovery of ribosome-associated complexes. After translation at 37°C for 60 min, reactions were treated with or without MNase and then loaded onto 10%–50% sucrose gradients. Gradients were centrifuged at 35 000 rpm for 3 h at 4°C and fractionated. Fractions corresponding to monosome- and disome-associated complexes were collected for downstream analysis. Proteins recovered from gradient fractions were analyzed by anti-V5 immunoblotting as described below. RNA recovered from the corresponding fractions was analyzed by denaturing urea-PAGE or anti-ADPr immunoblotting where indicated.

### Western blotting

Western blotting was performed to detect FLAG- and V5-tagged reporter proteins from bacterial cell lysates, *in vitro* translation reactions, and sucrose gradient fractions. Protein samples were mixed with SDS loading buffer and boiled at 95°C for 5–10 min. After boiling, samples were briefly centrifuged at maximum speed in a benchtop centrifuge, and the supernatants were loaded onto SDS–PAGE gels. Proteins were separated by electrophoresis and transferred to membranes using a Mini Trans-Blot electrophoretic transfer system. Membranes were blocked in Tris-buffered saline containing 0.1% Tween 20 (TBST) supplemented with 5% non-fat milk for 60 min at room temperature with gentle shaking. Primary antibodies against FLAG or V5 were diluted in blocking buffer and incubated with the membranes overnight at 4°C with gentle shaking. Membranes were washed with TBST and incubated with HRP-conjugated secondary antibodies diluted in blocking buffer for 1 h at room temperature. After washing with TBST, blots were developed using Clarity Max ECL substrate and visualized using a ChemiDoc imaging system.

For cellular reporter assays, *E. coli* strains carrying the indicated reporter constructs were grown under the indicated induction or non-induction conditions. Cells were harvested by centrifugation, and pellets were resuspended in phosphate buffered saline (PBS) or SDS loading buffer. Samples were normalized by culture density, mixed 1:1 with 2× SDS loading buffer, boiled at 95°C for 10 min, and clarified by centrifugation before loading. Anti-FLAG immunoblotting was used to detect C-terminally FLAG-tagged reporter products.

For *in vitro* translation assays, PURExpress reactions programmed with the indicated mRNA templates were incubated as described earlier. Aliquots of the total reactions were mixed with SDS loading buffer, boiled at 95°C for 5 min, and loaded directly onto SDS–PAGE gels. Anti-FLAG immunoblotting was used to detect C-terminally FLAG-tagged full-length reporter products, whereas anti-V5 immunoblotting was used to detect N-terminally V5-tagged translation products.

For sucrose-gradient fraction immunoblotting, proteins were recovered from individual fractions after TRIzol LS extraction as described earlier. Protein pellets were washed, resuspended in SDS loading buffer, boiled at 95°C for 5 min, and loaded onto SDS–PAGE gels. Anti-V5 immunoblotting was used to detect N-terminally V5-tagged translation products recovered from gradient fractions.

### Anti-ADPr immunoblotting

RNA used for anti-ADPr immunoblotting was isolated from sucrose gradient fractions of *in vitro* translations using TRIzol LS reagent according to the manufacturer’s instructions. Purified RNA was separated on an 8% urea-PAGE gel and then transferred onto Zeta-probe quaternary amine nylon membranes (Bio-Rad) using the Mini Trans-Blot electrophoretic transfer system (Bio-Rad). After transfer, RNA was crosslinked to the membrane by UV irradiation at 120 mJ/cm^2^. Membranes were blocked for 1 h at room temperature in blocking buffer (1× PBS, 0.05% Tween-20 w/v, 0.2% I-block reagent). Membranes were then incubated overnight at 4°C with monoclonal poly/mono-ADP-ribose antibody (E6F6A; Cell Signaling Technology) diluted 1:5000 in fresh blocking buffer. After washing with PBS-T, membranes were incubated for 1 h at room temperature with HRP-conjugated anti-rabbit secondary antibody diluted 1:5000 in blocking buffer. Blots were washed with PBS-T, developed using Clarity Max ECL substrate (Bio-Rad), and imaged using a ChemiDoc imaging system (Bio-Rad).

### Quantitative real-time RT-PCR analysis

To determine the expression of the genes in toxin-induced and control conditions, total RNA was isolated using the Monarch Spin RNA Isolation Kit (NEB) according to the manufacturer’s instructions. Subsequently, total RNA was reverse transcribed using random primers and LunaScript RT SuperMix (NEB) according to the manufacturer’s instructions. qPCR was carried out with Luna Universal qPCR Master Mix on a Real-Time PCR system (Bio-Rad), following the manufacturer’s recommendations. The 16S rRNA gene was used as the endogenous reference control, and relative gene expression levels were calculated using the comparative threshold cycle 2^−∆∆CT^ method.

To assess reverse-transcriptase read-through *in vitro*, reporter RNA was ADP-ribosylated by RhsP2 and purified as described earlier. cDNA synthesis was initiated using a gene-specific primer annealing to the 3′-terminal region of the RNA, allowing reverse transcription to proceed toward the 5′ end and cross the modification-containing region. The resulting cDNA was quantified by qPCR using two primer pairs: an upstream read-through amplicon and a downstream control amplicon. Read-through efficiency was evaluated by calculating the cycle threshold difference ∆Ct = Ct_upstream_ − Ct_downstream_ between the two amplicons. The primers for qPCR are listed in Supplementary Table S3.

### Visualization of crosslinked RNA–protein complexes by biotin labeling

To validate the recovery of RhsP2*-associated RNA, on-bead 3′ end biotinylation and streptavidin blotting were performed. Briefly, RhsP2*-3×FLAG or 3×FLAG control complexes were immunoprecipitated from UV-crosslinked lysates using Anti-FLAG magnetic beads. Where indicated, the bead-bound complexes were subjected to limited RNase I digestion (40 U) to fragment the associated RNA.

Following RNase I treatment and stringent washing, the bead-bound RNA fragments were treated with T4 Polynucleotide Kinase (PNK, NEB) to coordinate the RNA ends for ligation. The associated RNA was then 3′ end labeled on-beads with pCp-biotin using T4 RNA Ligase 1 (NEB). The reaction was incubated at 16°C overnight with gentle agitation.

After washing to remove unincorporated pCp-biotin, the protein–RNA complexes were eluted from the beads by boiling in SDS–PAGE loading buffer. The samples were resolved by SDS–PAGE and transferred to nitrocellulose membranes. Biotin-labeled RNA was visualized using the Pierce™ Chemiluminescent Nucleic Acid Detection Module (Thermo Fisher Scientific) following the manufacturer’s instructions. Membranes were blocked, incubated with Streptavidin-HRP conjugate, detected using enhanced chemiluminescence (ECL) substrate, and visualized using a ChemiDoc Imager.

### eCLIP-seq library preparation

eCLIP experiments were performed according to previously described standard operating procedures with minor modifications [41, 42]. Briefly, *E. coli* cells harboring pSCrhaB2-CV::RhsP2*-FLAG or pSCrhaB2-CV::3 × FLAG were grown in LB medium at 37°C to an OD_600_ of ~0.4. RhsP2*-3 × FLAG or 3 × FLAG expression was induced by addition of 0.2% L-rhamnose for 20 min. Cells were then transferred to culture dishes and irradiated with 400 mJ/cm^2^ UV light at 254 nm to crosslink RNA–protein complexes. Cells were harvested by centrifugation at 5000 rpm for 10 min at 4°C, washed or resuspended in 10 mM Tris–HCl, pH 8.0, and lysed in 1 ml CLIP lysis buffer containing 50 mM Tris–HCl, pH 7.4, 100 mM NaCl, 1% NP-40, 0.1% SDS, 0.5% sodium deoxycholate, and 1:200 protease inhibitor cocktail III.

Lysates were subjected to limited RNase I digestion for 5 min at 37°C, and RhsP2*–RNA complexes were immunoprecipitated overnight at 4°C using Anti-FLAG M2 Magnetic Beads. Beads were washed stringently with wash buffer containing 20 mM Tris–HCl, pH 7.4, 10 mM MgCl_2_, and 0.2% Tween-20. Bead-bound RNA was dephosphorylated with FastAP Thermosensitive Alkaline Phosphatase for 15 min at 37°C, phosphorylated with T4 polynucleotide kinase for 20 min at 37°C, and ligated on beads to a barcoded 3′ RNA adapter using T4 RNA ligase in the presence of PEG8000. On-bead ligation allowed removal of unincorporated adapter by subsequent washing. RhsP2*–RNA complexes were separated by SDS–PAGE and transferred to nitrocellulose membranes. Membrane regions corresponding to RhsP2*–RNA complexes were excised, and RNA was recovered by proteinase K digestion. Purified RNA was reverse transcribed, and excess oligonucleotides were removed using AMPure XP beads. A second adapter containing a 10-nt random barcode was ligated to the cDNA fragments, followed by bead cleanup, PCR amplification, agarose gel size selection, and final purification. Libraries were sequenced on an Illumina HiSeq 4000 platform.

### Rifampicin-chase RNA-seq (RIF-seq) library preparation and sequencing

RIF-seq was performed as previously described [35]. Briefly, empty vector control and RhsP2 expressing *E. coli* were grown under the indicated induction conditions before transcription was arrested by addition of rifampicin to a final concentration of 800 µg/ml. Samples were collected at 0, 2, 6, and 12 min after rifampicin addition and immediately mixed with two volumes of RNAlater solution. Samples were incubated at room temperature for 5 min to stabilize RNA, centrifuged at 12 000 rpm for 5 min at 4°C, and the resulting cell pellets were flash-frozen in liquid nitrogen and stored at −80°C until RNA extraction.

Total RNA was isolated using a Monarch Spin RNA Isolation Kit (NEB) according to the manufacturer’s instructions. For each sample, 1 µg of total RNA was used for library preparation. rRNA was depleted using the NEBNext rRNA Depletion Kit, and sequencing libraries were prepared using the NEBNext Ultra II Directional RNA Library Prep Kit for Illumina (NEB). All rRNA depletion and library preparation steps were performed according to the manufacturer’s instructions. Library quality was assessed using an Agilent 4200 TapeStation with D1000 ScreenTape, and library concentration was measured using a Qubit 4 Fluorometer with the Qubit 1× double-stranded DNA (dsDNA) HS Assay Kit. Libraries were sequenced by Novogene on an Illumina NovaSeq X Plus platform using paired-end 150-bp reads.

### Library preparation of monosome- and disome-seq libraries

Ribosome profiling was performed as described with minor modification [36, 37]. Single colonies were inoculated into LB medium and grown overnight at 37°C with shaking. The next day, overnight cultures were diluted 1:100 into 100 ml fresh LB and grown to an OD_600_ of 0.4. Expression of RhsP2 or the corresponding empty-vector control was induced by addition of L-rhamnose to a final concentration of 0.2% for 20 min. Cultures were rapidly chilled on ice for 5 min, and cells were harvested by vacuum filtration and immediately lysed in ice-cold lysis buffer containing 20 mM Tris–HCl, pH 8.0, 10 mM MgCl_2_, 100 mM NH_4_Cl, 5 mM CaCl_2_, 100 U/ml DNase I, and 1 mM chloramphenicol. Lysates were clarified by centrifugation at 20 000 × *g* for 15 min at 4°C. A260 absorbance of the lysate was measured, and lysate corresponding to 12 AU of RNA was digested with 800 U MNase in a final reaction volume of 400 µl at room temperature for 15 min.

For separation of ribosomal complexes, 10%–50% sucrose density gradients were prepared using a Gradient Master in gradient buffer. Digested lysates were loaded onto the gradient and centrifuged in an SW41 rotor at 35 000 rpm for 3 h at 4°C. Gradients were fractionated and monosome and disome peaks were collected as indicated.

RNA was extracted from collected monosome and disome fractions using TRIzol LS reagent according to the manufacturer’s instructions. RNA fragments were resolved on denaturing TBE–urea polyacrylamide gels alongside size markers. For monosome-seq libraries, ribosome-protected fragments (RPFs) of ~20–30 nt were excised and gel-purified. For disome-seq libraries, longer protected fragments of ~40–60 nt were excised and gel-purified. Excised gel fragments were crushed and incubated in 600 µl RNA elution buffer overnight at 4°C with rotation. Recovered RPFs were purified and treated with T4 polynucleotide kinase to generate ligation-competent RNA termini before library construction. Sequencing libraries were prepared using the NEBNext Low-bias Small RNA Library Prep Kit according to the manufacturer’s instructions. Final library quality was assessed using an Agilent 4200 TapeStation with D1000 ScreenTape, and libraries were quantified using a Qubit 4 Fluorometer with the Qubit 1× dsDNA HS Assay Kit. Libraries were sequenced on an Illumina NovaSeq 6000 platform using single-end 150-cycle reads.

### Analysis of eCLIP-seq data

Raw eCLIP-seq reads were trimmed to remove adapter and randomized barcode sequences, followed by quality and length filtering. Low-quality bases with Phred quality scores below 20 were trimmed and reads shorter than 20 nt after trimming were discarded. Reads were further removed if >30% of bases had Phred quality scores below 20. The resulting clean reads were mapped to the *E. coli* K-12 MG1655 reference genome using Bowtie2. PCR duplicates were collapsed using randomized barcode sequences and genomic mapping coordinates before peak calling.

RhsP2*-associated eCLIP-seq peaks were identified by comparing RhsP2*-3 × FLAG IP libraries with their corresponding input libraries. In parallel, 3 × FLAG IP and input libraries were processed as negative controls. Because the 3 × FLAG control libraries yielded very low numbers of clean reads and a low fraction of genome-mapped reads, they were not used for quantitative peak calling in downstream analyses. Candidate RhsP2*-associated peaks were defined from clusters of overlapping tags enriched in the RhsP2*-3 × FLAG IP relative to input and evaluated using a feature-level randomization procedure. For each gene or annotated feature, the observed maximum peak height was calculated from aligned eCLIP-seq tags. The same number of tags, while preserving the observed tag-length distribution, was randomly simulated across the corresponding gene or annotated feature, and the maximum random tag height generated by overlapping simulated tags was recorded. This procedure was repeated 500 times for each feature. Empirical *P* values were estimated as the fraction of simulations in which the maximum random tag height was equal to or greater than the observed peak height. Candidate peaks with empirical *P* < .01 were retained, whereas peaks failing this threshold were removed. Retained peaks were normalized and annotated according to genomic features for downstream analyses. Motif enrichment analysis was performed using HOMER. Enriched motifs identified from RhsP2*-specific peaks were used for downstream motif-centered analyses.

### Analysis of ribosome profiling libraries

Monosome and disome ribosome-profiling libraries were processed using a standardized ribosome-profiling analysis workflow adapted from previously established bacterial Ribo-seq methods [37, 38]. Briefly, sequencing reads were demultiplexed and trimmed to remove adapter sequences and randomized barcode sequences using Cutadapt v4.1. Reads shorter than 20 nt or with Phred quality scores below 20 were discarded. The resulting clean reads were sequentially filtered by mapping to rRNA and tRNA reference sequences, and reads derived from rRNAs or tRNAs were removed from downstream analyses. The remaining reads were aligned to the *E. coli* K-12 MG1655 reference CDSs using Bowtie2, allowing up to two mismatches.

For monosome profiling, reads corresponding to canonical monosome-protected footprints, typically 25–30 nt in length, were retained for downstream analyses. Genes with fewer than 0.5 reads per nucleotide on average were excluded from analysis. On each gene, codons close to the ends of the gene were likewise excluded (27 nt downstream of the start codon and 12 nt upstream of the stop codon). To calculate pause scores, we normalized the read count at each nt of a gene by dividing by the mean read count for the gene. For each codon, we calculated the mean value including reads from all three nt. Average pause scores were calculated using these values from all instances of the codon or amino acid of interest. Pause scores calculated for the *P* site used a −14 nt offset from the 3′end of monosome footprints.

To assess RhsP2-dependent changes in ribosome occupancy, CDSs passing the coverage filters were divided into 10 equal-length bins, and *P*-site distributions were compared between control and RhsP2-induced samples using an overdispersion-adjusted Pearson chi-square test. *P* values were corrected using the Benjamini–Hochberg method, with adjusted *P* ≤ .05 considered significant.

For disome profiling, longer RPFs, typically 40–60 nt in length, were selected as disome-protected footprints. For motif-centered endpoint analyses, disome reads were not collapsed to a single inferred ribosomal position; instead, the 5′ and 3′ extremities of disome-protected fragments were analyzed separately to capture collision-associated footprint geometry. Differential disome signals were calculated by comparing normalized coverage between RhsP2-induced and vector control samples. For each position, differential *Z*-score signal was calculated as the difference between RhsP2-induced and control *Z*-normalized coverage values. Heatmaps and average profiles were generated by aligning disome footprint extremities to motif-centered positions and plotting signal across the indicated window relative to the motif anchor.

### Analysis of RIF-seq RNA half-life analysis library

Raw sequencing reads were first subjected to quality control using fastp v0.24.0. Reads with an average Phred quality score below 20 were filtered, and reads shorter than 20 nt after trimming were discarded. Paired-end adapter sequences were automatically detected and removed by fastp, generating clean reads for downstream analysis. Quality-filtered clean reads were aligned to the *E. coli* K-12 MG1655 reference genome (GCA_000 005 845), downloaded from Ensembl, using Bowtie2 v2.5.4 in paired-end mode. Bowtie2 genome indices were generated using bowtie2-build. Alignment files were converted to BAM format, sorted, and indexed using SAMtools v1.21. Gene-level read counts were quantified from sorted BAM files using featureCounts from the Subread package. Reads were counted at the CDS and ncRNA feature levels. Raw count matrices were normalized as TPM values to generate comparable abundance estimates across samples.

RNA decay was modeled using a first-order exponential decay model, consistent with standard RNA kinetic frameworks in which RNA degradation follows first-order kinetics [35, 39]:


\begin{eqnarray*}
E\left( t \right) = A \cdot {{e}^{ - kt}},
\end{eqnarray*}

where *E*(*t*) represents the normalized RNA abundance at time *t, A* represents the fitted abundance at *t* = 0, and *k* is the first-order degradation rate constant. For each CDS in the empty-vector control and RhsP2-expressing conditions, normalized abundance values from the 0, 2, 6, and 12 min time points after rifampicin treatment were fitted independently using the nls() function in R. Nonlinear least-squares regression was performed using the Gauss–Newton algorithm to minimize the residual sum of squares.

For CDSs with fitted *k* > 0, RNA half-lives were calculated as


\begin{eqnarray*}
{{t}_{\frac{1}{2}}} = \frac{{ln2}}{k}.
\end{eqnarray*}

CDSs for which the model failed to converge, yielded a non-positive decay constant, or produced fitted half-life estimates beyond the predefined upper limit, consistent with essentially non-decaying behavior within the experimental time window, were excluded from downstream half-life distribution analyses. Final CDS-level half-life values for the empty-vector and RhsP2-expressing conditions were visualized using custom scripts.

### Quantification and statistical analysis

Statistical analyses are described in the corresponding figure legends and method details, including the statistical tests used, replicate numbers, and definitions of significance. Details of data processing, quantification, normalization, transcript stratification, and software used are provided in the relevant method details sections.

## Results

### RhsP2 preferentially associates with mRNAs

To determine whether mRNAs function as physiological substrates of RhsP2, we performed enhanced UV cross-linking and immunoprecipitation coupled with high-throughput sequencing (eCLIP-seq) [40–42] in *E. coli* expressing 3 × FLAG-tagged, catalytically inactive RhsP2 (RhsP2^E1576Q^; henceforth, RhsP2*) (Fig. 1A). We previously established that low-copy expression of RhsP2 in *E. coli* recapitulates the RNA ADP-ribosylation observed during RhsP2-mediated interbacterial competition [30], supporting the use of this system to investigate physiological RhsP2 targets. We employed the catalytically inactive variant because active RhsP2 is expected to interact only transiently with RNA substrates during catalysis, whereas we reasoned that RhsP2* would retain the ability to bind RNA without turning over substrate. Consistent with this prediction, RhsP2* bound a model RNA substrate with affinity comparable to that of WT RhsP2 measured under NAD⁺-free conditions (apparent *K*_d_ ≈ 7.2 µM) (Supplementary Fig. S1A). To confirm that the 3 × FLAG tag itself did not impair toxin activity, we compared the effects of tagged WT and catalytically inactive RhsP2 on bacterial growth. Whereas 3 × FLAG-tagged WT RhsP2 retained growth-inhibitory activity, 3 × FLAG-tagged RhsP2* did not (Supplementary Fig. S1B). Anti-FLAG immunoprecipitation of UV-crosslinked lysates efficiently enriched RhsP2*-3 × FLAG (Supplementary Fig. S1C and D), and associated RNAs were subsequently 3′-end labeled with pCp-biotin and detected by streptavidin blotting, revealing a high molecular weight smear consistent with enrichment of covalently trapped RhsP2*–RNA complexes suitable for downstream eCLIP-seq analysis (Supplementary Fig. S1C–E). Together, these experiments support the use of RhsP2* as a binding-competent, catalytically inactive proxy for transcriptome-wide identification of candidate RhsP2 targets.

eCLIP-seq analysis identified numerous RhsP2*-bound peaks enriched over size-matched input controls, as illustrated by representative genome browser tracks (Fig. 1B). Replicate level correlation analyses confirmed the reproducibility of the eCLIP-seq dataset (Supplementary Fig. S2A–F). Consistent with our previous findings, a subset of peaks localized to non-coding RNAs, including sRNAs and tRNAs [26, 30]. However, most RhsP2*-associated peaks mapped to mRNA CDSs (Fig. 1C). Metagene analysis supported this distribution, revealing enrichment throughout coding regions with minimal signal in the upstream and downstream flanking regions (Supplementary Fig. S3A). *De novo* motif discovery identified recurrent UAA-centered sequence features, including a highly enriched UAAG-containing motif, among RhsP2*-associated RNAs (Fig. 1D and Supplementary Fig. S3B and C). Together, these data indicate that RhsP2* preferentially associates with UAA enriched sites within mRNA CDSs.

To directly test whether the motif enrichment observed in our eCLIP-seq data reflects an intrinsic contribution of the local RNA sequence, we performed targeted biochemical assays *in vitro*. Using the same catalytically inactive RhsP2* variant employed for eCLIP-seq, we first assessed binding to a 25-nt RNA containing the WT UAAG motif and a series of progressively altered sequence variants (Fig. 1E). EMSAs showed that disruption of the UAAG-centered sequence (UACG, CACG, and CCCG) generally reduced apparent binding relative to the UAAG-containing substrate (Fig. 1F, top).

We next asked whether this sequence preference also influences the efficiency of RhsP2-catalyzed RNA modification. Under deliberately limiting reaction conditions (0.1 μM WT RhsP2 for 10 s), the UAAG-containing RNA was efficiently ADP-ribosylated, whereas progressive disruption of the motif markedly reduced modification (Fig. 1F, bottom). Time-course analyses performed using a 10-fold higher RhsP2 concentration (1 μM) further revealed a clear hierarchy of modification efficiency, with the UAAG-containing substrate undergoing the most rapid and efficient modification among the tested variants (Supplementary Fig. S4A and B). Under these more extensive reaction conditions, additional slower-migrating modified species became apparent, consistent with modification occurring at multiple sites within individual RNA substrates. This observation aligns with previous biochemical analyses establishing that RhsP2 exhibits broad activity toward structured RNA substrates and can modify multiple RNA nucleotides [30]. Consistent with these observations, our findings indicate that UAAG-containing motifs are kinetically preferred targets of RhsP2-catalyzed RNA ADP-ribosylation, even though additional sites can be modified within the same RNA molecule.

To more precisely map the modification region, we took advantage of the guanosine at the 3′ end of the UAAG sequence, which provides a diagnostic RNase T1 cleavage site (Fig. 1E). ADPr-RNA products were subjected to AH, using RNase T1 digestion as a nucleotide reference ladder. Compared with unmodified RNA, ADPr-RNA products exhibited protection from NaOH-induced cleavage within the UAAG motif (Fig. 1G). Because RhsP2-catalyzed ADP-ribosylation occurs on ribose 2′-hydroxyl groups [30], protection from AH is consistent with modification of ribose 2′-hydroxyl groups within this region, as alkaline RNA cleavage requires nucleophilic attack by the ribose 2′-OH on the adjacent phosphodiester bond to generate a 2′,3′-cyclic phosphate intermediate [43, 44]. Across motif variants, substitutions that impaired RhsP2-catalyzed ADP-ribosylation restored NaOH-induced cleavage at the protected position (Supplementary Fig. S4C). Together, these mapping and biochemical data support a model in which RhsP2 preferentially targets ribose 2′-hydroxyl groups within UAAG-enriched sequence contexts while still modifying multiple positions within RNA substrates.

### RhsP2 uncouples mRNA stability from productive translation

Having shown that RhsP2 associates with UAA-enriched sites within mRNAs *in vivo* and ADP-ribosylates UAA-containing RNA with enhanced kinetics *in vitro*, we next asked how RhsP2 activity alters mRNA stability. To address this, we measured transcriptome-wide mRNA decay following rifampicin-mediated transcriptional arrest in control and RhsP2-induced cells using rifampicin-chase RNA-seq (RIF-seq) (Fig. 2A). Biological replicates showed high pairwise correlations and clustered by RhsP2 induction condition and time point, supporting the reproducibility of the RIF-seq dataset (Supplementary Fig. S5A). In the control strain, the median mRNA half-life was 6.2 min, consistent with previous transcriptome-wide measurements in *E. coli*, where typical mRNA half-lives range from 2–7 min [35, 45–47]. In contrast, RhsP2 induction increased the median mRNA half-life to ~52 min, indicating widespread stabilization of cellular mRNAs (Fig. 2B and Supplementary Fig. S5B). Cumulative distribution and density analyses similarly revealed a pronounced rightward shift in transcript half-lives following RhsP2 induction (Supplementary Fig. S5C). Together, these findings indicate that RhsP2 broadly suppresses mRNA decay and drives the accumulation of unusually stable mRNAs across the bacterial transcriptome.

**Figure 2.**
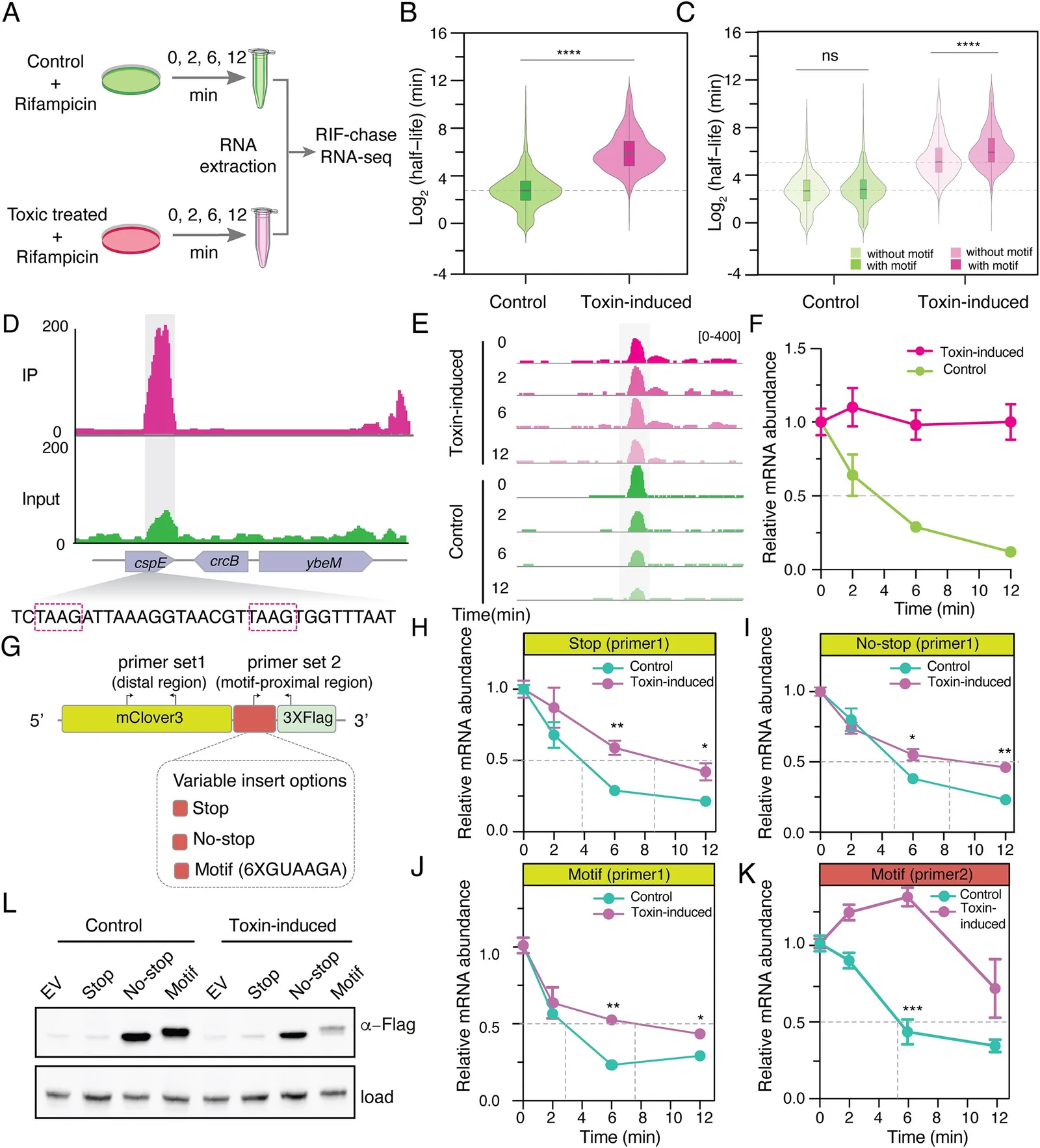
RhsP2 stabilizes target mRNAs while uncoupling transcript persistence from productive protein synthesis. (**A**) Workflow for transcriptome-wide mRNA half-life measurements by rifampicin-chase RNA-seq (RIF-seq). Following RhsP2 induction or empty-vector control treatment, transcription was inhibited with rifampicin, and cells were harvested at 0, 2, 6, and 12 min for RNA extraction, RNA-seq, and half-life estimation. *n* = 3 biological replicates. (**B**) Genome-wide distribution of mRNA half-lives in control and RhsP2-induced cells. Half-lives are plotted as log_2_-transformed values. Statistical significance was assessed using a two-sided Wilcoxon rank-sum test within each condition; ^****^P ≤ .0001. (**C**) Motif-stratified analysis of transcriptome-wide mRNA half-lives. Transcripts were grouped according to the presence or absence of UAAG motif within CDSs, and transcript half-lives were compared between groups. Statistical comparisons are indicated. Statistical significance was assessed using a two-sided Wilcoxon rank-sum test within each condition. ns, not significant; ^****^P ≤ .0001. (**D**) Genome-browser view of the cspE locus showing RhsP2* eCLIP-seq enrichment in the IP sample relative to matched input. UAAG-related motif occurrences (shown as TAAG in DNA notation) are boxed. cspE was selected as a representative RhsP2-associated transcript for independent validation. (**E**) RNA-seq coverage tracks across the cspE locus during the rifampicin-chase time course. Tracks are shown at 0, 2, 6, and 12 min after rifampicin addition for control and RhsP2-induced cells. (**F**) qRT-PCR validation of cspE mRNA decay following rifampicin treatment. Relative cspE mRNA levels are shown over time in control and RhsP2-induced cells. (**G**) Schematic of the tagged reporter used to measure regional differences in RhsP2-dependent RNA stabilization and protein output. Reporter constructs contain a variable insert comprising a stop sequence, a no-stop control sequence, or a UAAG motif cassette upstream of a C-terminal 3 × FLAG tag. Primer set 1 interrogates the upstream mclover3 region distal to the variable insert, whereas primer set 2 interrogates the motif-proximal region. (H–J) Rifampicin-chase qRT-PCR validation of reporter mRNA decay using primer set 1, which detects the distal mclover3 region indicated in panel (G). Relative reporter mRNA levels are shown over time for transcripts containing a stop control (**H**), a no-stop insert (**I**), and 6 × GUAAGA motif cassette (**J**) under control and RhsP2-induced conditions. (**K**) Rifampicin-chase qRT-PCR analysis of the 6 × GUAAGA motif-containing reporter using primer set 2, which detects the motif-proximal region indicated in panel (G). Relative reporter mRNA abundance is shown over time under control and RhsP2-induced conditions. For panels (H–K), statistical significance was assessed using two-way analysis of variance (ANOVA) followed by Šídák’s multiple-comparisons test comparing control and RhsP2-induced conditions at each time point. *P ≤ .05, **P ≤ .01, ***P ≤ .001. (**L**) Anti-FLAG immunoblot analysis of reporter protein accumulation under control and RhsP2-induced conditions. A nonspecific band is shown below as a loading control.

We next examined whether RhsP2-dependent mRNA stabilization was associated with the highly enriched UAAG motif identified by eCLIP-seq. Transcripts were stratified according to the presence or absence of UAAG motifs within coding regions. Following RhsP2 induction, UAAG-containing transcripts had a significantly longer median half-life than transcripts lacking the motif (56 versus 38 min), whereas no significant difference was observed between the two groups under control conditions (Fig. 2C). These findings indicate that RhsP2-dependent stabilization is greater among UAAG-containing transcripts, consistent with preferential stabilization of RhsP2-targeted transcripts rather than a nonspecific consequence of toxin expression. Consistent with this interpretation, the high-confidence RhsP2 target *cspE*, which exhibited strong eCLIP-seq enrichment (Fig. 2D), underwent rapid decay in control cells but remained markedly stabilized following RhsP2 induction (Fig. 2E). qRT-PCR analysis independently confirmed delayed decay kinetics at this locus, with *cspE* transcript abundance remaining near baseline levels under RhsP2-induced conditions while rapidly decreasing in the control strain (Fig. 2F). Similar stabilization patterns were observed across additional RhsP2-associated transcripts (Supplementary Fig. S6), indicating that RhsP2-dependent mRNA stabilization is a general feature of targeted transcripts.

We next asked whether stabilized mRNAs remained competent for protein production. To examine this relationship in a defined system, we generated a series of tagged reporter constructs containing either a premature stop codon, no inserted motif, or a 6 × UAAG motif cassette positioned upstream of a C-terminal 3 × FLAG tag (Fig. 2G). We first examined whether RhsP2-dependent stabilization differed across regions of these transcripts using distinct qRT-PCR primer sets (Fig. 2G). qRT-PCR targeting the upstream *mclover3* region (Primer 1) showed modest stabilization of all three reporter transcripts following RhsP2 induction (Fig. 2H–J). For the 6 × UAAG-containing reporter, however, analysis with Primer 2, which targets the motif-proximal region, revealed substantially greater transcript persistence (Fig. 2K) than that observed in the distal *mclover3* region measured with Primer 1 (Fig. 2J). Thus, the distinct decay kinetics detected at different regions of the same reporter transcript indicate that RhsP2-dependent stabilization is preferentially associated with the UAAG-rich region rather than being uniformly distributed across the transcript. Despite this preferential stabilization of the UAAG-enriched region of the reporter mRNA, anti-FLAG immunoblotting revealed substantially reduced accumulation of FLAG-tagged reporter protein from the 6 × UAAG-containing reporter following RhsP2 induction, whereas the no-stop control reporter retained detectable protein accumulation (Fig. 2L). In the absence of RhsP2 induction, both reporters accumulated comparable levels of FLAG-tagged protein, indicating that the 6 × UAAG cassette itself does not intrinsically impair reporter expression (Fig. 2L). Thus, localized stabilization of the UAAG-enriched region of the transcript was accompanied by reduced production of the encoded FLAG-tagged protein following RhsP2 induction. Together, these findings demonstrate that RhsP2 uncouples mRNA stability from efficient translation.

### RhsP2-modified mRNAs impair translation elongation

The accumulation of stable but translationally unproductive transcripts prompted us to examine how ADP-ribosylated mRNAs affect translation *in vivo*. Given that RhsP2* eCLIP-seq peaks were predominantly localized within CDSs, we first asked whether RhsP2 activity prevents ribosome loading or instead impairs translation elongation. Polysome profiling showed that overall polysome architecture was broadly preserved following toxin induction (Fig. 3A and B), arguing against a dominant global defect in ribosome recruitment or translation initiation. Moreover, denaturing urea-PAGE analysis of RNA recovered from monosome fractions revealed distinct slower-migrating RPFs specifically in RhsP2-induced samples (Fig. 3C and D, red dots), consistent with ADP-ribosylated RPFs being retained within ribosome-protected material.

**Figure 3.**
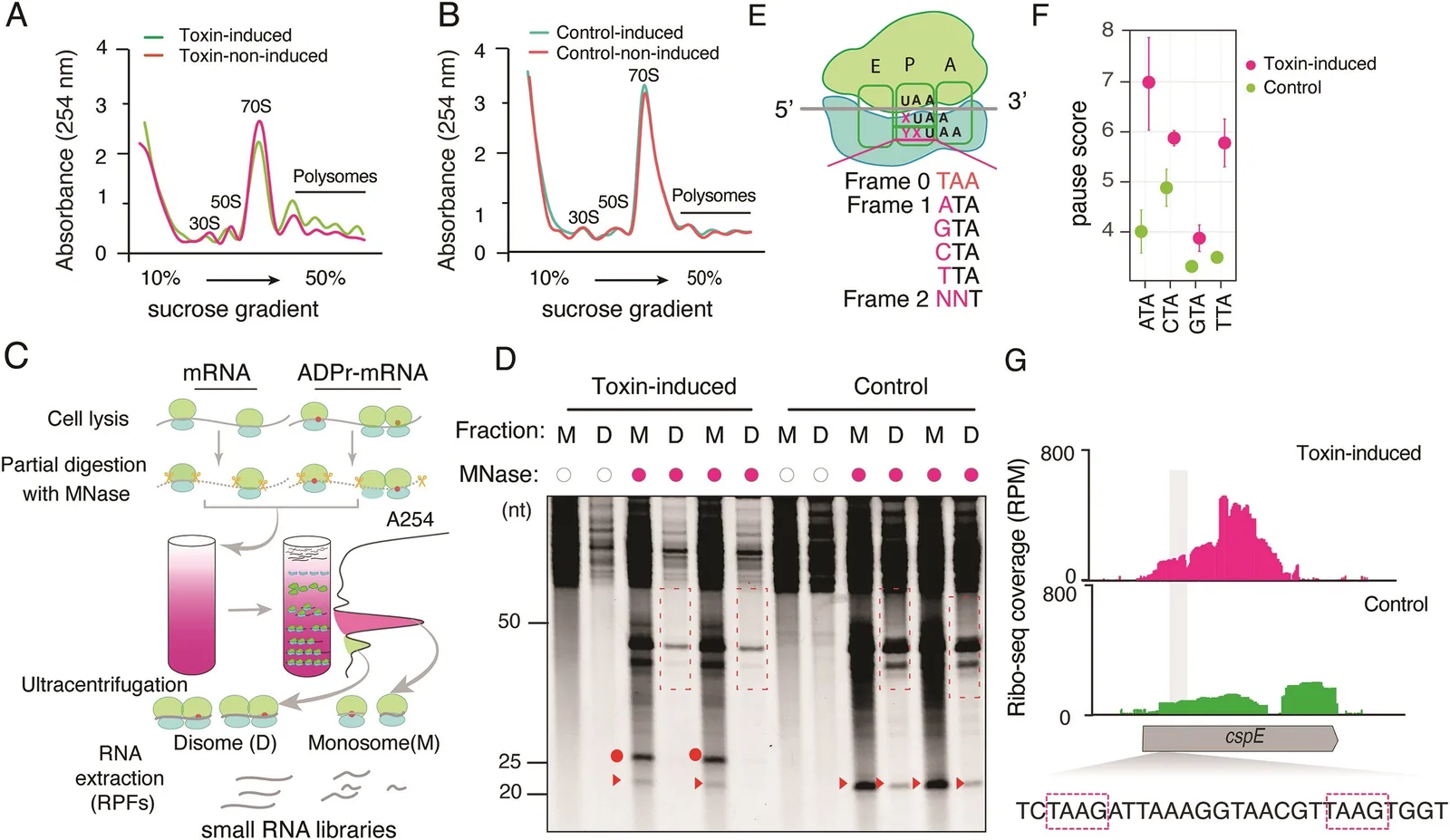
RhsP2 induces ribosome pausing during translation elongation. Representative sucrose-gradient A254 profiles of RhsP2-induced (**A**) or control (**B**) cells showing 30S, 50S, 70S, and polysome fractions. (**C**) Schematic of the monosome and disome footprinting workflow following MNase digestion and sucrose-gradient fractionation. Monosome (M) and disome (D) fractions were collected for RPF isolation and sequencing. (**D**) Denaturing urea-PAGE analysis of RPFs recovered from monosome (M) and disome (D) fractions. Samples from control and RhsP2-induced cells were analyzed with or without MNase treatment as indicated. Two biological replicates are shown for each condition. Red triangles indicate canonical monosome-sized RPFs, whereas red circles indicate slower-migrating RPF species, and dashed boxes indicate the RNA size ranges excised for library preparation. (**E**) Top: schematic representation of a UAA-containing sequence positioned within the ribosomal P-site. Bottom: codons generated from the UAA motif in different reading frames. (**F**) Codon-level pause scores for UAA-derived codon contexts in control and RhsP2-induced cells. Pause scores are elevated in RhsP2-induced samples across multiple motif-derived codon classes. (**G**) Representative genome-browser view of mono-seq coverage across the cspE locus in RhsP2-induced and control cells. Boxed TAAG sequences indicate UAAG-containing sites in the corresponding RNA transcript.

To map ribosome occupancy at higher resolution, we isolated RPFs from monosome fractions for deep sequencing (mono-seq) (Fig. 3C). Although RhsP2-induced samples contained a slower-migrating RPF population by denaturing urea-PAGE (Fig. 3D), the corresponding sequencing libraries retained the expected 26–30 nt footprint length distribution observed in control libraries (Supplementary Fig. S7A). The altered electrophoretic mobility therefore does not reflect an increase in footprint length and is instead consistent with ADP-ribosylation of ribosome-protected RNA fragments, indicating that RhsP2-induced RPFs remain suitable for downstream mapping of ribosome occupancy. We next mapped ribosome occupancy relative to UAAG-centered sites across the transcriptome. For metagene analysis, RPF coverage was aligned to the first adenosine of the UAAG motif (position 0), corresponding to the motif-proximal ADP-ribosylation site inferred from biochemical mapping. This analysis revealed a significant enrichment of RPF signal centered on the UAAG motif in RhsP2-induced cells that was largely absent from control samples (Supplementary Fig. S7B). The focal nature of this enrichment suggests that RhsP2-catalyzed ADP-ribose modifications create localized barriers to ribosome progression rather than reflecting nonspecific global changes in translation.

To quantify ribosome pausing at the codon level, we calculated transcriptome-wide P-site pause scores for all 61 sense codons in both control and RhsP2-induced samples [38, 48]. Because UAAG motifs can occur in different reading frames within CDSs, the same motif can give rise to distinct codon identities in the translated frame (Fig. 3E). Stop codons were excluded from the analysis because RPF accumulation at translation start and stop sites reflects normal initiation and termination events rather than internal elongation pausing [37, 38, 48]. P-site pause scores were elevated in RhsP2-induced samples for several codons generated from UAAG-containing motifs, including the frame + 1 codons ATA, CTA, GTA, and TTA, with the strongest effects observed for ATA, CTA, and TTA (Fig. 3F). A similar, although more broadly distributed, increase was also observed for codons arising from the frame +2 interpretation of the motif (NNT codons), with CTT, AGT, ACT, and GAT among the codons showing increased pause scores following RhsP2 induction (Supplementary Fig. S7C). Thus, the pausing signature tracks with the underlying UAAG motif rather than a specific codon identity, indicating that RhsP2-induced RNA modifications create localized barriers to translation elongation.

To determine whether these RhsP2-dependent changes extended beyond a limited number of representative transcripts, we performed a gene-level analysis of P-site positional distributions across the transcriptome. Among 402 genes analyzed, 394 showed significant RhsP2-dependent changes in ribosome occupancy distribution (Supplementary Fig. S7D). UAAG-containing genes showed stronger statistical evidence of RhsP2-dependent ribosome redistribution than genes lacking the motif (Wilcoxon rank-sum test, *P* = 0.00384; Supplementary Fig. S7E). A weaker association was observed for UACG (*P* = 0.02166), whereas CACG and CCCG were not significantly associated (*P* = 0.09396 and 0.1678, respectively; Supplementary Fig. S7F–H). Together, these analyses show that RhsP2-dependent ribosome redistribution is most strongly associated with the preferred UAAG sequence context. At the individual transcript level, the representative high-confidence RhsP2 target *cspE* displayed pronounced ribosome footprint enrichment over regions containing UAAG-centered sites following RhsP2 induction compared with the control (Fig. 3G). Together, these transcriptome-wide and transcript-level analyses support a model in which RhsP2-induced mRNA modifications create localized barriers to translation elongation at coding region target sites.

### RhsP2-induced mRNA ADP-ribosylation promotes ribosome collisions

We next asked whether RhsP2-induced ribosome stalls are sufficiently persistent to impede trailing ribosomes and generate ribosome collisions. Disome profiling provides a transcriptome-wide readout of bacterial ribosome collisions at translational stall sites [49]. To test whether RhsP2-induced stalling generates collided ribosome complexes *in vivo*, we performed sucrose-gradient profiling of ribosome-associated material from RhsP2-induced and control cells, with or without micrococcal nuclease (MNase) treatment. In the absence of nuclease digestion, polysome-associated material was readily detected in both conditions, consistent with ongoing translation (Fig. 4A and B). Following MNase treatment, exposed mRNA between translating ribosomes is expected to be degraded, whereas closely packed collided ribosomes can remain protected as disome-sized complexes [50]. Consistent with collision formation, a disome-associated peak remained evident in RhsP2-induced samples following MNase treatment, whereas the corresponding feature was less apparent in control samples (Fig. 4A and B). Quantification of peak areas by trapezoidal integration showed preferential retention of the disome-associated signal following MNase treatment in RhsP2-induced samples, whereas monosome peak areas were comparatively maintained or increased (Fig. 4C). Together, these findings indicate that a subset of RhsP2-induced elongation stalls is sufficiently persistent to generate nuclease-resistant collided ribosome complexes.

**Figure 4.**
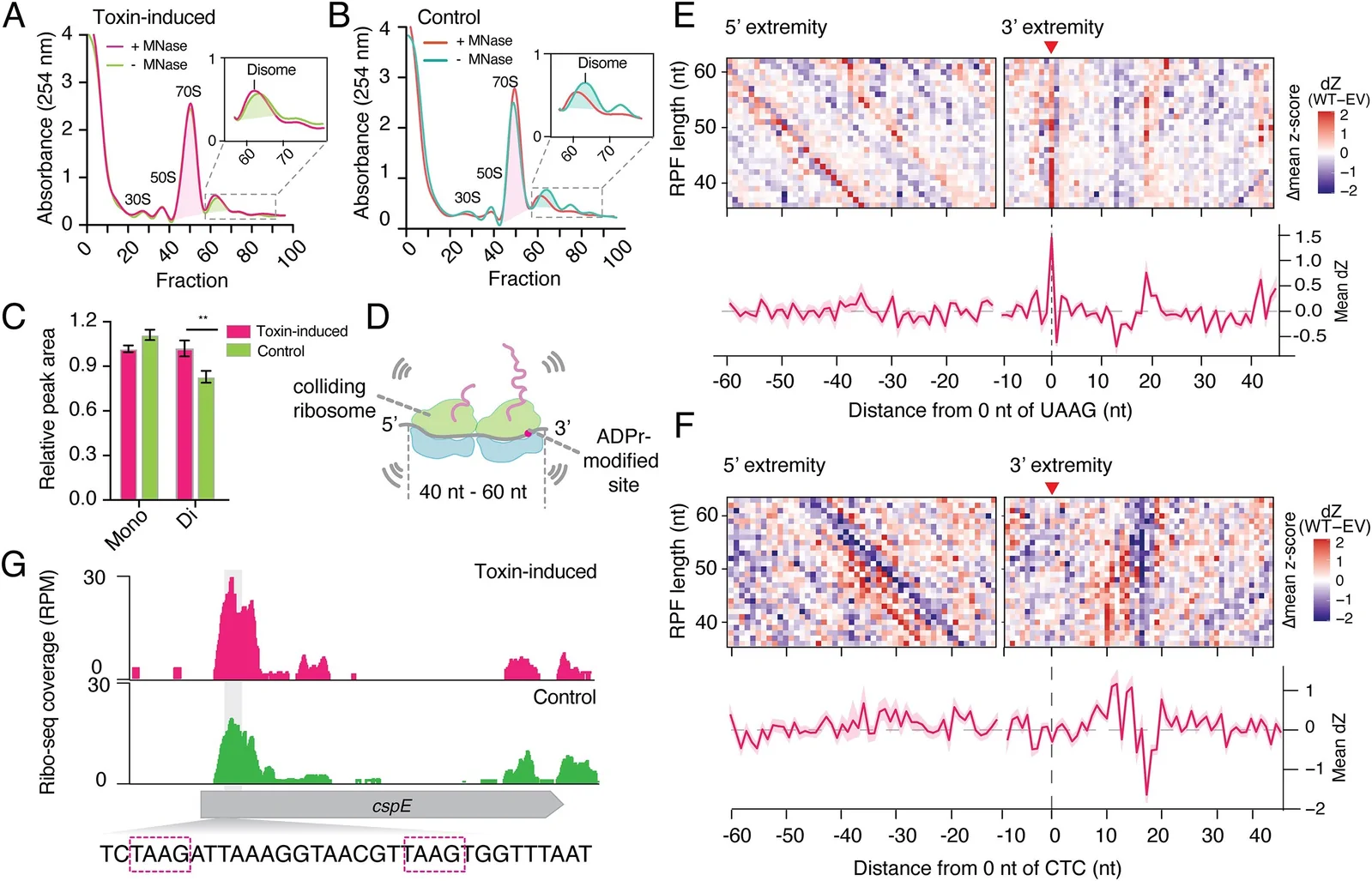
RhsP2-induced stalls promote ribosome collisions. Sucrose-gradient profiles of ribosome-associated complexes from RhsP2-induced cells (**A**) and control cells (**B**), analyzed with or without MNase treatment. Absorbance at 254 nm is plotted across gradient fractions. Insets show zoomed-in views of the disome-associated regions indicated by the dashed boxes. (**C**) Quantification of monosome and disome peak areas from sucrose-gradient profiles in RhsP2-induced and control samples. Peak areas were normalized within each condition to enable comparison of monosome- and disome-associated fractions. Data are shown as mean ± SD from independent biological replicates. Statistical significance was assessed by two-way ANOVA followed by Sidak’s multiple-comparisons test. **P ≤ .01. (**D**) Model of ribosome collision formation on an ADP-ribosylated mRNA. A leading stalled ribosome and a trailing ribosome form a collided disome complex. Disome-seq metaprofiles aligned to the first adenosine of TAAG sites (**E**) or the first cytosine of sequence-matched CTC sites (**F**), each defined as position 0. Heatmaps show differential *Z*-score signal (dZ = RhsP2-induced – control) for the 5′- and 3′-extremities of disome-protected fragments across disome-sized read lengths. Arrowheads indicate the anchor position used for alignment. Traces below each heatmap show the mean dZ signal averaged across read lengths at each relative position. (**G**) Representative genome-browser view of disome-seq footprint coverage across the cspE locus in RhsP2-induced and control samples. Boxed regions indicate TAAG sites within the displayed region.

This result predicts that ribosome collision events should localize to RhsP2-associated sequence contexts. To test this, we defined the positions of collided ribosome complexes across the transcriptome by isolating disome-protected fragments and performing disome-seq (Fig. 3C and D). To identify collision events specifically associated with RhsP2 induction, we calculated differential *Z*-score (dZ) signals between RhsP2-induced and control samples and aligned the 5′ and 3′ extremities of disome footprints relative to motif-centered sites (Fig. 4D-F). At UAAG-centered sites, alignment to the first adenosine of the motif (position 0) revealed localized positive dZ enrichment near the anchor position. Average dZ profiles likewise showed a focal increase in disome endpoint signal centered on the motif (Fig. 4E), indicating that RhsP2-induced ribosome collision events preferentially accumulate near UAAG-centered target sites.

To assess whether this motif-centered collision signal was specific to the preferred UAAG sequence context, we extended the same analysis to the UACG, CACG, and CCCG sequence variants, as well as to CTC sites used as a non-target reference (Supplementary Fig. S8 and Fig. 4F). Compared with the focal enrichment observed at UAAG-centered sites, the average dZ profiles for these non-UAAG sequence contexts showed generally weaker and less coherent motif-proximal enrichment. These results support a preferential association between the UAAG sequence context and RhsP2-dependent ribosome collision rather than a general consequence of local sequence composition or read-mapping bias.

Finally, we examined individual target transcripts to determine whether the global disome-seq pattern could be observed at specific loci. At the representative target gene *cspE*, RhsP2 induction produced a strong localized ribosome occupancy peak over a motif-associated region, whereas the corresponding signal was markedly weaker in control samples (Fig. 4G). Together, the sucrose-gradient profiling, motif-centered disome-seq, and transcript-level analyses support a model in which RhsP2-induced mRNA ADP-ribosylation promotes ribosome collisions at coding-region target sites.

### ADP-ribosylated mRNAs are sufficient to block productive translation *in vitro*

To determine whether RhsP2-mediated mRNA ADP-ribosylation directly impedes translation, we used the 6 × UAAG reporter described previously as a defined substrate for *in vitro* translation assays. Reporter mRNAs encoding a dual-tag reporter protein were purified and either left unmodified or ADP-ribosylated by RhsP2 prior to translation (Fig. 5A). In this system, anti-FLAG detects full-length translation products whereas anti-V5 detects both full-length and arrested N-terminal products. As a positive control, we included SecM, which contains a well-characterized C-terminal arrest sequence (FSTPVWISQAQGIRAGP) that stalls bacterial ribosomes through interactions of the nascent peptide with the ribosomal exit tunnel [51, 52].

**Figure 5.**
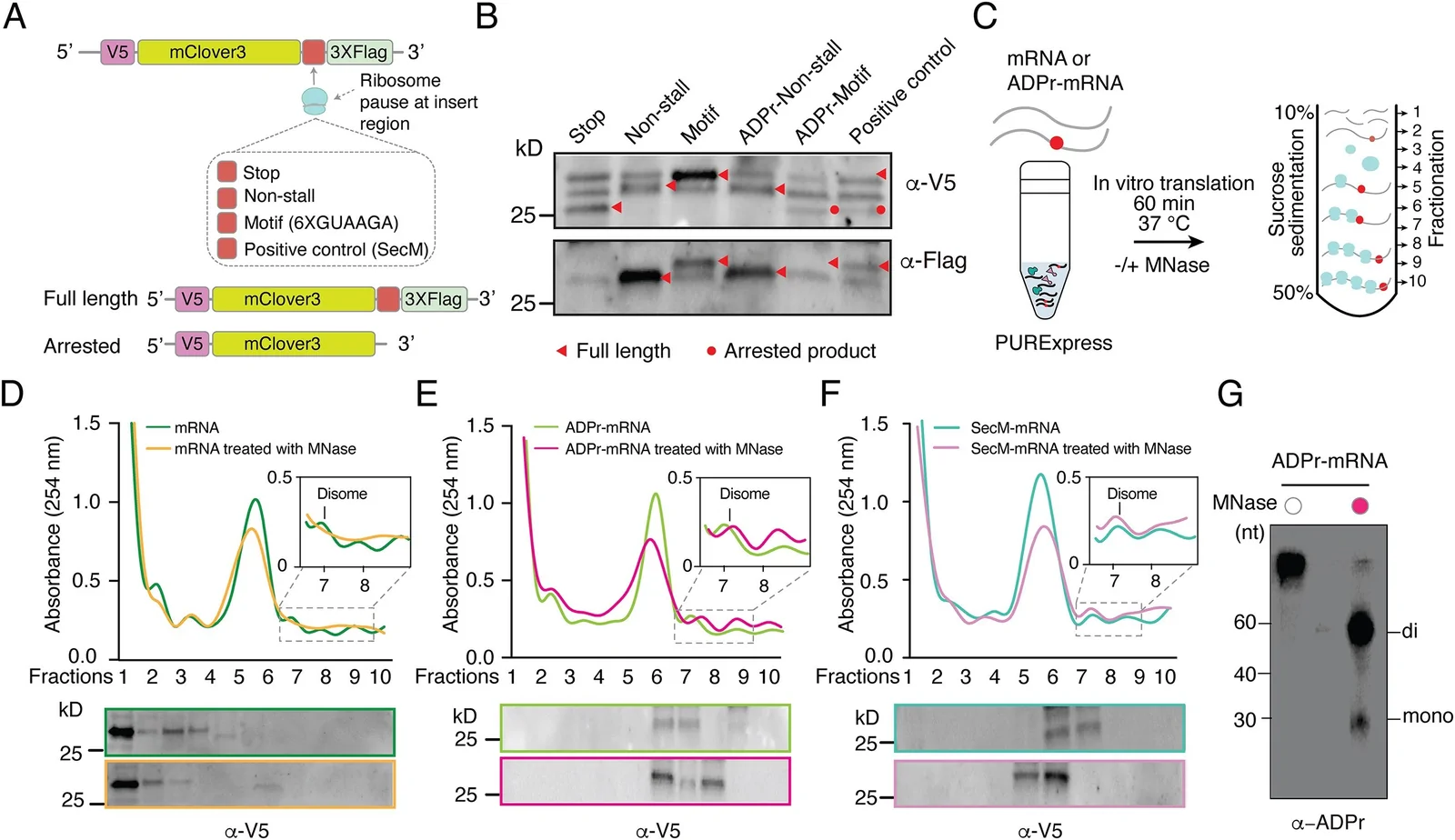
ADP-ribosylated mRNA blocks productive translation *in vitro*. (**A**) Schematic of the dual-tag reporter used to monitor translation completion *in vitro*. The reporter contains an N-terminal V5 tag, mClover, a variable insert region, and a C-terminal 3 × FLAG tag. Full-length translation generates V5- and FLAG-positive products, whereas translational arrest upstream of the C-terminal tag generates V5-positive, FLAG-negative products. As a positive control for translational arrest, the variable insert region contained the C-terminal SecM arrest peptide sequence FSTPVWISQAQGIRAGP. (**B**) Anti-V5 and anti-FLAG immunoblots of *in vitro* translation products generated from the indicated reporter RNAs. Red arrowheads indicate the expected primary translation products; red circles indicate arrested or truncated products. (**C**) Workflow for *in vitro* translation and ribosome complex fractionation. Unmodified or ADP-ribosylated reporter mRNAs were translated using the PURExpress system at 37°C for 60 min, treated with or without MNase, separated on 10%–50% sucrose gradients, and analyzed following fraction collection. A254 sucrose gradient profiles (upper) and corresponding anti-V5 immunoblots of gradient fractions (lower) from translation reactions containing unmodified 6 × UAAG-containing RNA (**D**), ADP-ribosylated 6 × UAAG-containing RNA (**E**), or SecM arrest peptide-encoding control RNA (**F**), with or without MNase treatment. Insets show zoomed-in views of the disome-associated regions indicated by the dashed boxes. (**G**) Anti-ADPr immunoblot analysis of purified RNA recovered from monosome- and disome-associated fractions following *in vitro* translation and MNase treatment.

As expected, unmodified 6 × UAAG-containing mRNA supported full-length protein synthesis, indicating that the motif cassette itself does not prevent productive translation (Fig. 5B). By contrast, ADP-ribosylation of the 6 × UAAG-containing mRNA nearly abolished the C-terminal FLAG signal and generated V5-positive arrested products similar to the SecM control (Fig. 5B). ADP-ribosylated control mRNA retained detectable full-length translation and did not generate the same arrested-product pattern. These data indicate that mRNA ADP-ribosylation is sufficient to block translation elongation while still allowing translation initiation.

We next asked whether this translational arrest promotes the formation of collided ribosome complexes. Translation reactions were separated on sucrose gradients with or without MNase treatment (Fig. 5C). In reactions containing unmodified 6 × UAAG-containing mRNA, MNase treatment reduced the disome-associated peak, indicating that higher-order ribosome complexes on this template were not stably protected from nuclease digestion (Fig. 5D). In contrast, reactions containing ADP-ribosylated motif-containing mRNA retained a prominent MNase-resistant disome peak (Fig. 5E). A similar nuclease-resistant species was observed with the stalled SecM peptide control RNA (Fig. 5F), consistent with the formation of collided ribosome complexes during elongation arrest.

Immunoblot analysis of the corresponding gradient fractions further supported this conclusion. V5-positive arrested products were enriched in ribosome-associated fractions from ADP-ribosylated and SecM-containing reactions, consistent with the accumulation of incomplete nascent-chain products within stalled ribosome complexes [52, 53]. Anti-ADPr immunoblotting of RNA recovered from the same fractions detected ADPr-positive species (Fig. 5G), directly linking modified mRNA to the protected ribosome complexes formed *in vitro*. Together, these findings indicate that ADP-ribosylated mRNAs remain competent for ribosome loading but create a barrier to productive elongation, resulting in the accumulation of MNase-resistant, collision-associated ribosome complexes.

### RhsP2-induced translational stress engages bacterial ribosome rescue pathways

The accumulation of stalled and collided ribosomes on ADP-ribosylated mRNAs suggested that RhsP2 intoxication imposes a substantial burden on bacterial ribosome quality-control systems. In *E. coli*, persistent elongation stalls are resolved primarily through tmRNA-mediated *trans*-translation, which rescues arrested ribosomes and promotes degradation of incomplete nascent polypeptides [18, 33, 53, 54]. We therefore examined whether ribosome rescue pathways become engaged during RhsP2-induced translational stress.

Previous work identified the tmRNA as a target of RhsP2-mediated ADP-ribosylation [30]. Consistent with a functional connection between RhsP2 and the *trans*-translation machinery, genome-browser analysis revealed strong enrichment of RhsP2 eCLIP-seq signal over the *ssrA/tmRNA* locus (Fig. 6A). In parallel, ribosome profiling showed a marked increase in RPF coverage over the tmRNA mRNA-like domain (MLD), which serves as the coding template during *trans*-translation. Quantification revealed an approximately two-fold increase in the fraction of ribosome footprints mapping to the MLD following toxin induction (Fig. 6A and B). Together, these findings demonstrate that RhsP2 expression is associated with pronounced perturbation of the tmRNA pathway and increased ribosome occupancy within the *trans*-translation template region.

**Figure 6.**
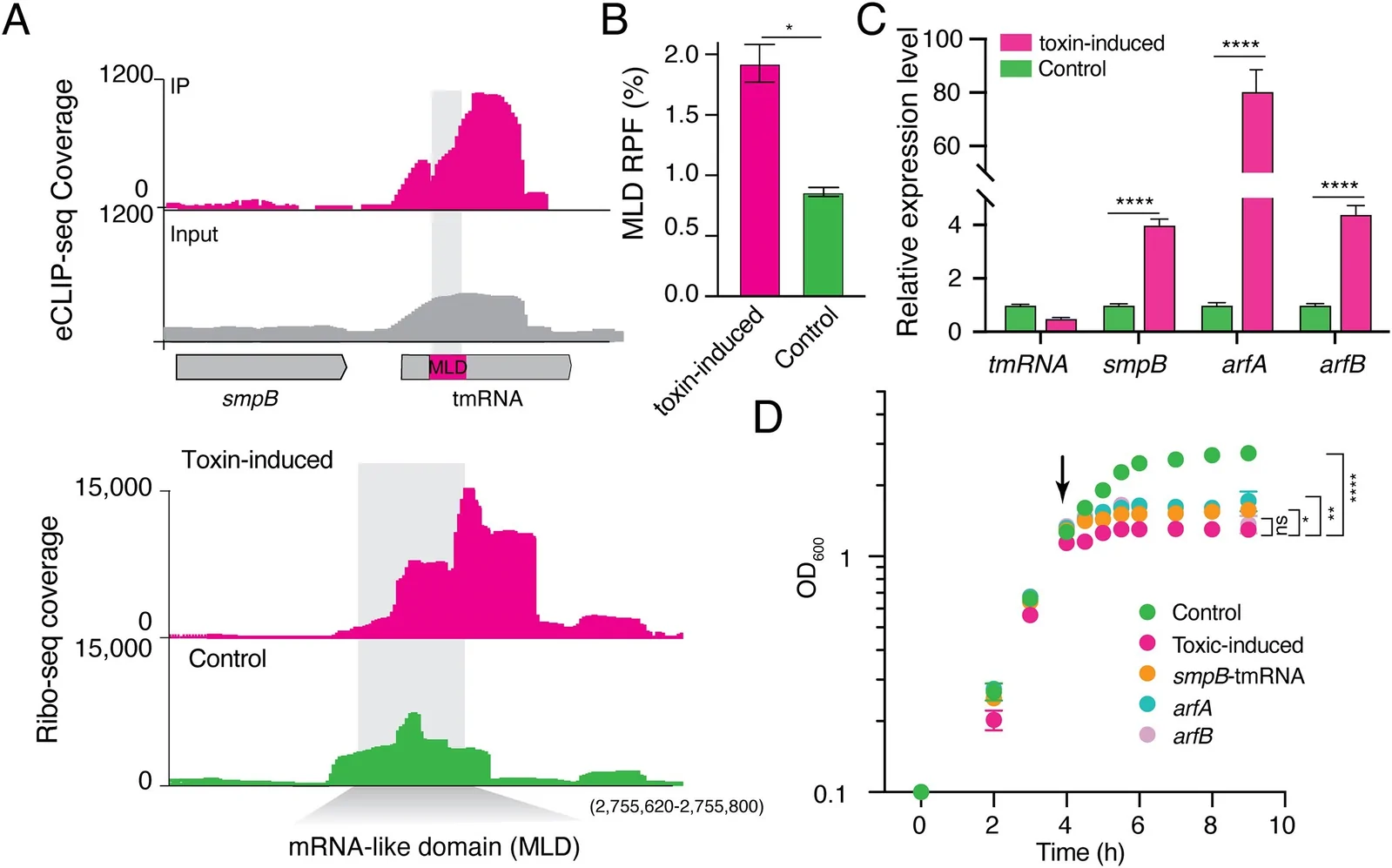
RhsP2 intoxication is associated with ribosome rescue pathway responses. (**A**) Genome-browser view of the smpB-ssrA/tmRNA locus showing RhsP2 eCLIP-seq signal and ribosome profiling coverage. Upper tracks show RhsP2 eCLIP-seq IP and input signals. Lower tracks show ribosome profiling coverage in RhsP2-induced and control cells. The tmRNA MLD is highlighted. (**B**) Quantification of RPFs mapping to the tmRNA MLD in RhsP2-induced and control cells. Data are shown as mean ± SD from independent biological replicates. Statistical significance was assessed using Student’s *t*-test comparing toxin-induced and control samples within each fraction; *P ≤ .05. (**C**) Relative transcript abundance of ribosome rescue factors in RhsP2-induced and control cells. Expression levels of smpB, arfA, and arfB were determined by qRT-PCR. Data are shown as mean ± SD from three biological replicates. Statistical significance was assessed by two-way ANOVA followed by Tukey’s multiple-comparisons test comparing control and RhsP2-induced samples within each gene. ns, not significant; ^****^P ≤ .0001. (**D**) Growth of cells expressing RhsP2 in the presence or absence of tmRNA–SmpB, ArfA, or ArfB overexpression. Data are shown as mean ± SD from three biological replicates. Statistical comparisons at the 9-h endpoint were performed using ordinary one-way ANOVA followed by Dunnett’s multiple-comparisons test, with RhsP2 WT as the reference group. Exact adjusted *P* values are indicated in the figure.

We next examined whether this translational stress was accompanied by broader changes in ribosome rescue systems. Quantitative RT-PCR revealed increased abundance of *smpB, arfA*, and *arfB* transcripts following RhsP2 induction, with *arfA* exhibiting the strongest response (Fig. 6C). Because SmpB functions together with tmRNA during *trans*-translation, whereas ArfA and ArfB provide alternative pathways for rescuing arrested ribosomes [33, 55], accumulation of these transcripts is consistent with increased demand on cellular ribosome rescue mechanisms during RhsP2 intoxication.

To determine whether enhancing rescue capacity could alleviate RhsP2-mediated toxicity, we monitored growth in cells expressing additional copies of tmRNA-SmpB or ArfA. As previously established, RhsP2 induction strongly inhibits bacterial growth [30]. Overexpression of either tmRNA-SmpB or ArfA modestly alleviated the RhsP2-induced growth defect, whereas ArfB overexpression had no significant effect (Fig. 6D), indicating that increasing ribosome rescue capacity might mitigate the consequences of RhsP2 intoxication. However, neither condition restored growth to control levels, demonstrating that rescue pathway overexpression is insufficient to fully counteract toxin-mediated growth inhibition.

Together, these findings indicate that RhsP2-mediated intoxication places substantial pressure on bacterial ribosome quality-control systems. Increased tmRNA-associated ribosome occupancy, accumulation of transcripts encoding canonical and alternative rescue factors, and partial suppression of toxicity by tmRNA-SmpB or ArfA collectively support a model in which persistent translation arrest disrupts cellular ribosome homeostasis. Whether direct modification of tmRNA further contributes to this phenotype remains an important question for future investigation.

## Discussion

Our study identifies mRNAs as a major physiological substrate of the RNA-targeting ADP-ribosyltransferase toxin RhsP2 and reveals an unexpected consequence of toxin-mediated RNA modification. Rather than promoting RNA degradation, RhsP2 converts targeted transcripts into stable but translationally inactive molecules. This state contrasts with the canonical relationship between mRNA abundance and protein production in bacteria, demonstrating that transcript persistence and translational competence can be separated through covalent modification of mRNA. Together, these findings establish mRNA ADP-ribosylation as a mechanism of translational inhibition that functionally uncouples mRNA stability from protein production.

The substrate landscape of RhsP2 highlights both shared and distinct features among the emerging class of RNA-targeting ADP-ribosyltransferases. Like the anti-phage toxin CmdT, RhsP2 also targets cellular mRNAs and inhibits protein synthesis through ADP-ribosylation. However, the two toxins differ substantially in substrate recognition and modification site. CmdT preferentially modifies adenines within GA-containing single-stranded RNAs through N6-linked ADP-ribosylation [32], whereas RhsP2 preferentially associates with UAA-enriched transcripts and catalyzes ribose 2′-OH ADP-ribosylation. Unlike an absolute sequence determinant, however, UAA sequence context enhances the kinetics of RNA modification while acting together with RNA structure to promote productive substrate recognition. Together, these findings suggest that mRNA ADP-ribosylation may be a more broadly employed strategy for translational inhibition than previously appreciated.

The most striking consequence of RhsP2 intoxication is the stabilization of transcripts that are no longer capable of supporting productive translation. In bacteria, transcript abundance and protein synthesis are generally closely linked, in part because translating ribosomes protect mRNAs from degradation whereas poorly translated transcripts are often rapidly turned over [17, 56–58]. Consistent with this paradigm, perturbations that impair translation frequently accelerate mRNA decay [53, 59]. RhsP2 instead produces the opposite outcome. These findings reveal that covalent modification of protein-coding transcripts can fundamentally alter the relationship between translation and transcript turnover.

The mechanisms responsible for transcript stabilization remain unclear. One possibility is that RhsP2-modified transcripts are stabilized through sustained ribosome occupancy rather than enhanced intrinsic resistance of the RNA itself. Ribosome occupancy is an important determinant of bacterial mRNA turnover, and extensive ribosome loading can protect transcripts by limiting access of ribonucleases to the underlying RNA [17, 60, 61]. Consistent with this model, RhsP2-modified transcripts remain associated with translating ribosomes and accumulate stalled and collided ribosome complexes. Together with the recovery of ADP-ribosylated RPFs, these observations suggest that the primary defect is not ribosome recruitment but productive elongation through modified transcripts. Thus, unlike many situations in which defective translation promotes mRNA decay [17, 61], RhsP2-mediated modification may stabilize transcripts by converting them into ribosome-shielded, translationally silent substrates.

Additionally, stabilization may arise in part from the chemistry of the ADP-ribose modification itself. RhsP2 modifies ribose 2′-hydroxyl groups, which contribute to RNA reactivity and influence interactions with RNA-processing enzymes. Because alkaline RNA cleavage depends on nucleophilic attack by the ribose 2′-hydroxyl group, ADP-ribosylation is expected to alter this chemistry. Consistent with this prediction, ADPr-RNAs were protected from AH, confirming that the modification directly affects 2′-OH-dependent cleavage reactions. Because only a subset of ribose 2′-hydroxyl groups within each transcript is likely to be modified, however, generalized protection of the RNA backbone is unlikely to fully account for the pronounced stabilization observed *in vivo*. Instead, modification of a limited number of functionally important sites could disproportionately affect transcript turnover. RNase E, a major initiator of bacterial mRNA decay, preferentially cleaves accessible AU-rich sequence contexts that overlap with the UAA-enriched regions targeted by RhsP2. This overlap raises the possibility that ADP-ribosylation occurs at or near RNase E-sensitive sites, where modification could inhibit cleavage by altering substrate recognition or the local 2′-OH-dependent chemical environment. Whether these mechanisms contribute to transcript stabilization *in vivo* remains unclear, and direct comparison of RhsP2 modification sites with mapped RNase E cleavage sites will be required to test this model.

The stalled and collided ribosomes generated by RhsP2 are likely to place substantial demand on bacterial ribosome rescue pathways. In bacteria, stalled ribosomes are primarily resolved by tmRNA–SmpB-mediated *trans*-translation, whereas ArfA and ArfB provide alternative rescue routes when canonical rescue is insufficient or unavailable [34, 62–64]. Consistent with engagement of these pathways, RhsP2 induction increased ribosome occupancy over the MLD of tmRNA and elevated the abundance of transcripts encoding ribosome rescue factors, with *arfA* exhibiting the strongest response. Furthermore, increasing rescue capacity through overexpression of tmRNA-SmpB or ArfA partially alleviated toxin-mediated growth inhibition, indicating that ribosome rescue can mitigate the consequences of RhsP2 intoxication. Although neither condition fully restored growth, the incomplete rescue may reflect the widespread nature of RhsP2-induced ribosome stalling, additional toxic consequences of RNA modification, or the modification of tmRNA itself, which we previously identified as a substrate of RhsP2-mediated ADP-ribosylation [30], potentially limiting the benefit of tmRNA-SmpB overexpression.

Most characterized RNA-targeting toxins inhibit gene expression by promoting RNA degradation or by targeting essential structured RNAs. RhsP2 instead leaves transcripts intact but functionally compromised, allowing modified mRNAs to persist despite their inability to support productive translation. Taken together with the growing diversity of RNA-targeting ADP-ribosyltransferases acting on tRNAs, rRNAs, structured non-coding RNAs, and mRNAs, our findings demonstrate that RNA ADP-ribosylation represents a versatile strategy for biological conflict and reveal that covalent modification can uncouple mRNA stability from protein production without requiring RNA degradation.

## Supplementary Material

gkag1177_Supplemental_File

## Data Availability

The raw sequencing data generated in this study, including eCLIP-seq, monosome- and disome-seq, and rifampicin-chase RNA-seq, have been deposited in the NCBI Sequence Read Archive (SRA) under BioProject accession number PRJNA1473760. Processed sequencing data and genome-browser tracks are available through the Gene Expression Omnibus under accession number GSE346425. Original code generated for this work is available on GitHub (https://github.com/Guchen-Weng/halflife-calculation-ribo-seq.git) and Zenodo at https://doi.org/10.5281/zenodo.21783403. Additional information regarding the data in this study is available upon request to the corresponding author.
