# Supplementary material for: An RNA-targeting ADP-ribosyltransferase toxin uncouples mRNA stability from productive translation
Source: Nucleic Acids Res. 2026 Nov 14;54(21):gkag1177. doi: 10.1093/nar/gkag1177 (PMC13768812; doi:10.1093/nar/gkag1177)
Supplement: Supplementary file 1 [file gkag1177_supplemental_file.pdf]

An RNA-targeting ADP-ribosyltransferase toxin uncouples mRNA stability from productive translation

Fanyang Lv[1,2], Guchen Weng[2], and John C. Whitney[1,2,3*]

[1]Michael DeGroote Institute for Infectious Disease Research, McMaster University, Hamilton, Ontario L8S 4K1, Canada

[2]Department of Biochemistry and Biomedical Sciences, McMaster University, Hamilton, Ontario L8S 4K1, Canada

[3]David Braley Centre for Antibiotic Discovery, McMaster University, Hamilton, ON L8S 4K1, Canada

[*]To whom correspondence should be addressed: John C. Whitney, Department of Biochemistry and Biomedical Sciences, McMaster University, Hamilton, Ontario, L8S 4K1, Canada.

E-mail: jwhitney@mcmaster.ca

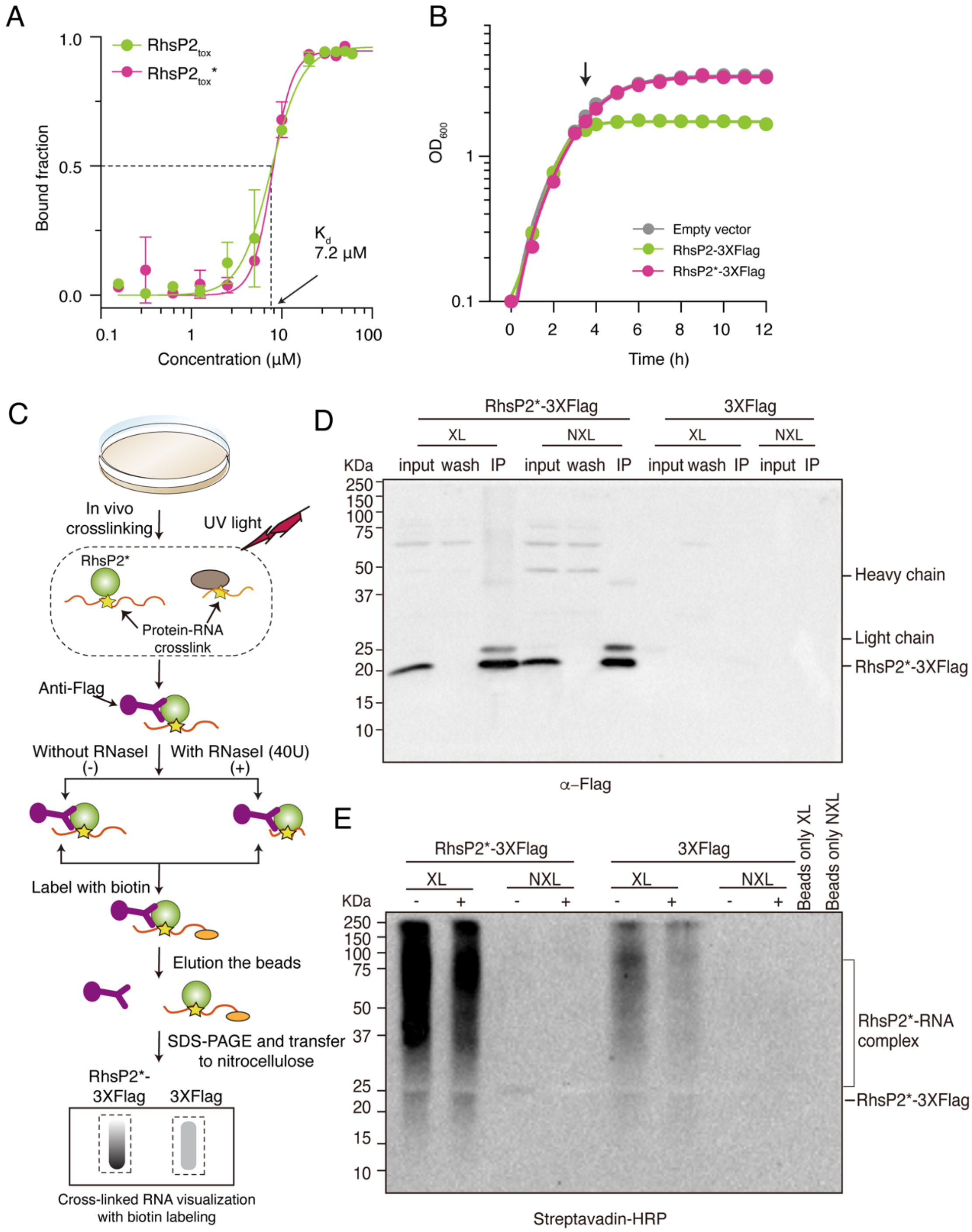

Figure S1. Validation of the catalytically inactive RhsP2 E1576Q variant for eCLIP-seq.

(A) Equilibrium binding of wild-type RhsP2 (green) and the catalytically inactive variant RhsP2* (E1576Q; magenta) to McaS RNA, measured by electrophoretic mobility shift

assay (EMSA). EMSA band intensities were quantified by densitometry and fitted to determine apparent dissociation constants (K_d). K_d values for RhsP2 and RhsP2* are indicated.

(B) Growth curves of *E. coli* expressing the indicated plasmids following induction with 0.2% L-rhamnose. The induction point is indicated by an arrow.

(C) Schematic of the UV-crosslinking and RNA recovery workflow used to validate RhsP2*–RNA complex formation. Cells expressing RhsP2*–3×FLAG were UV-crosslinked, lysed, and subjected to anti-FLAG immunoprecipitation. Associated RNAs were ligated to biotin at their 3′ ends, and RNA–protein complexes were resolved by SDS–PAGE, transferred to nitrocellulose membranes, and visualized by streptavidin blotting.

(D) Anti-FLAG immunoblot of input, wash, and immunoprecipitated (IP) fractions from crosslinked (XL) and non-crosslinked (NXL) RhsP2*–3×FLAG and vector control samples. IgG heavy- and light-chain bands are indicated.

(E) Streptavidin blot detection of biotin-labeled RNA–protein complexes recovered from crosslinked (XL) and non-crosslinked (NXL) RhsP2*–3×FLAG and 3×FLAG control samples. Samples were analyzed with (+) or without (−) RNaseI treatment as indicated. Beads-only controls are shown at right.

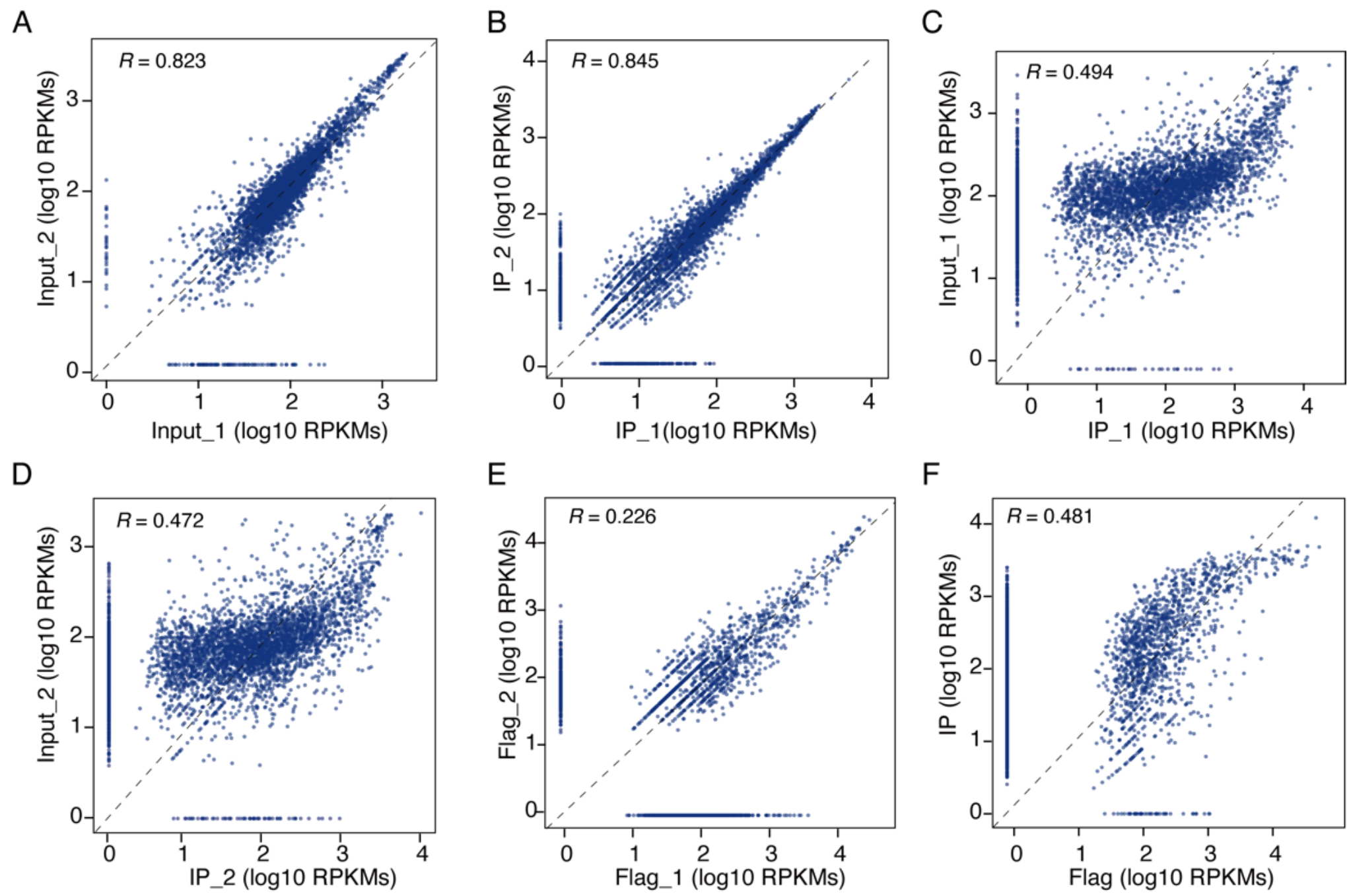

Figure S2. Reproducibility and specificity of RhsP2 eCLIP-seq datasets.
Scatter plots show peak-level eCLIP-seq signal quantified as $\log_{10}$(RPKM) for the indicated sample comparisons. Each point represents an individual peak. Dashed lines indicate the y = x diagonal. Pearson correlation coefficients (R) are shown in each panel.
(A) Input replicate concordance: Input 1 versus Input 2.
(B) IP replicate concordance: IP 1 versus IP 2.
(C) Enrichment relative to input: IP 1 versus Input 1.
(D) Enrichment relative to input: IP 2 versus Input 2.
(E) Vector control replicate concordance: Vector control 1 versus Vector control 2. The low correlation indicates weak and non-reproducible RNA recovery in the absence of RhsP2*, consistent with a lack of stable RNA enrichment by cells expressing the empty vector control.
(F) Comparison of pooled vector control IP and pooled RhsP2* IP datasets. The distinct signal distribution observed in the RhsP2* dataset compared to vector control supports the specificity of RhsP2-associated RNA enrichment.

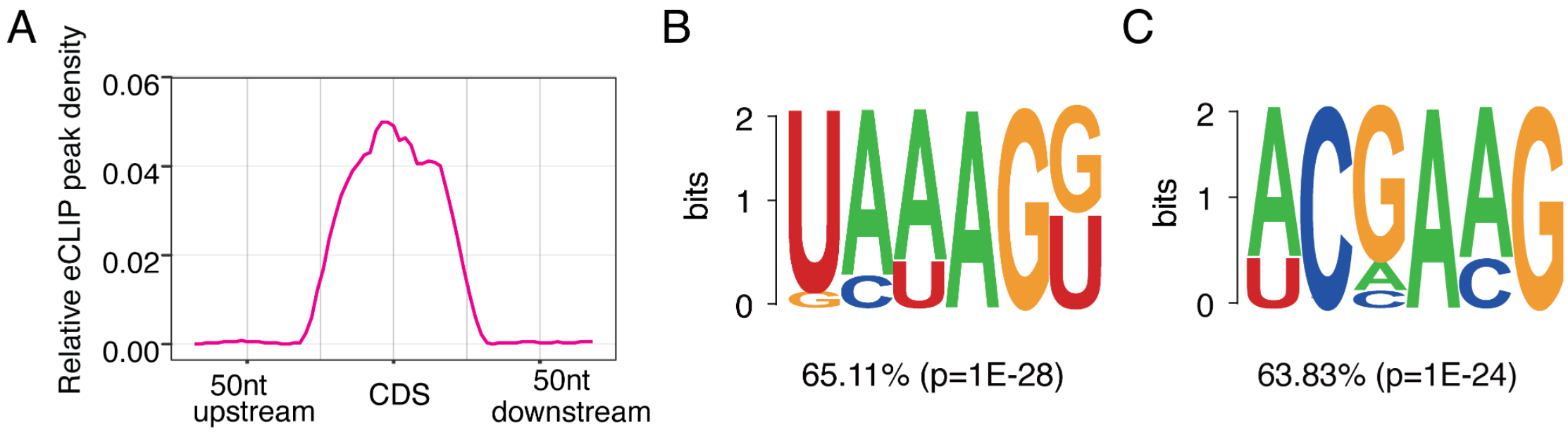

Figure S3. RhsP2 eCLIP peaks are enriched across coding regions and contain additional U/A-rich motifs.

(A) Metagene distribution of RhsP2* eCLIP peaks across mRNA regions. For each transcript, the 50-nt upstream region, coding sequence (CDS), and 50-nt downstream region were each divided into 50 scaled bins, and the fraction of eCLIP-seq peak summits falling within each bin was calculated. Relative peak density is plotted across the scaled transcript regions.

(B, C) Additional enriched motifs identified from RhsP2* eCLIP-seq peaks. Motif occurrence frequencies and enrichment P values are shown. The highest ranked motifs are presented in Figure 1D.

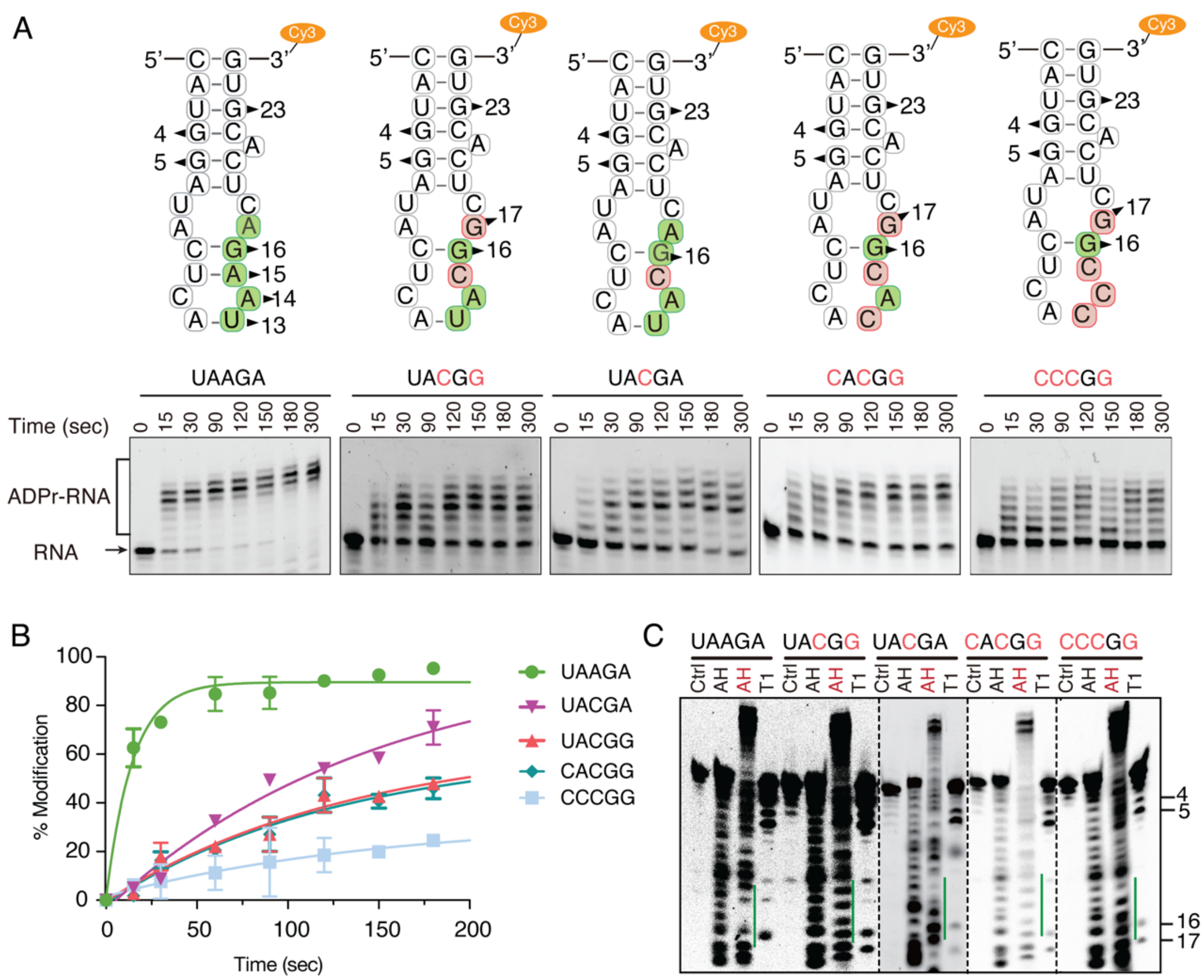

Figure S4. Kinetic and positional analysis of RhsP2 substrate preference.

(A) Time-course analysis of RhsP2-catalyzed RNA ADP-ribosylation. Cy3-labeled synthetic RNA substrates containing the indicated motif variants were incubated with 1mM NAD^+ and 1 µM RhsP2 for the indicated times, and reaction products were resolved by denaturing urea–PAGE. Unmodified RNA and slower-migrating ADP-ribosylated RNA (ADPr-RNA) species are indicated.

(B) Quantification of ADP-ribosylation kinetics from the time-course assays in (A). Modification levels were quantified by densitometry and plotted as the percentage of total RNA converted to ADPr-RNA over time. Curves were fitted to a single-exponential association model, $Y = Y_{max}(1 - e^{-kt})$.

(C) Alkaline hydrolysis and RNase T1 mapping of ADP-ribosylated RNA substrates containing the indicated motif variants. Unmodified RNA (Ctrl) and ADPr-RNA were subjected to alkaline hydrolysis (AH), and RNase T1 digestion was used as a nucleotide reference ladder. The red AH label denotes the alkaline hydrolysis lane generated from ADPr-RNA, and the green vertical line marks the UAAG motif region exhibiting protection from alkaline cleavage. Numbers indicate nucleotide positions within the

synthetic RNA substrate. The UAAGA-containing substrate corresponds to the consensus substrate shown in Figure 1F, G and is included here as a reference for comparison with the motif-variant substrates.

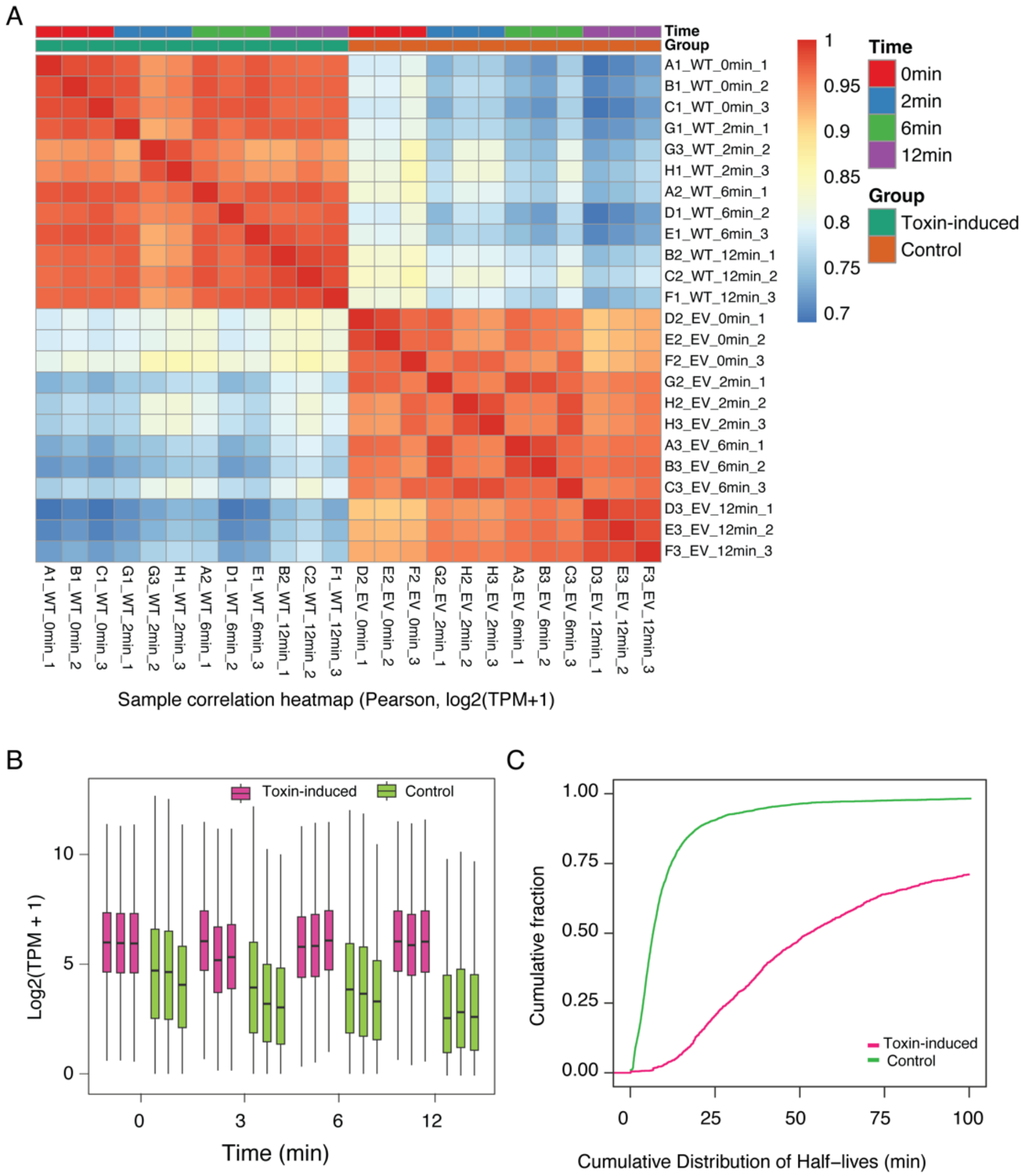

Figure S5. Quality control and global summary of the rifampicin-chase RNA-seq dataset used for mRNA half-life estimation.

(A) Sample–sample correlation matrix computed from gene-level expression values. Samples are annotated by condition and time point. Biological replicates show high concordance, and samples cluster primarily by condition across the rifampicin-chase time course.

(B) Distribution of gene-level expression values across time points and conditions. Expression is shown as $\log_2$(TPM + 1) for control and RhsP2-induced samples collected at 0, 2, 6, and 12 min after rifampicin addition.

(C) Cumulative distribution of transcript half-lives in control and RhsP2-induced samples.

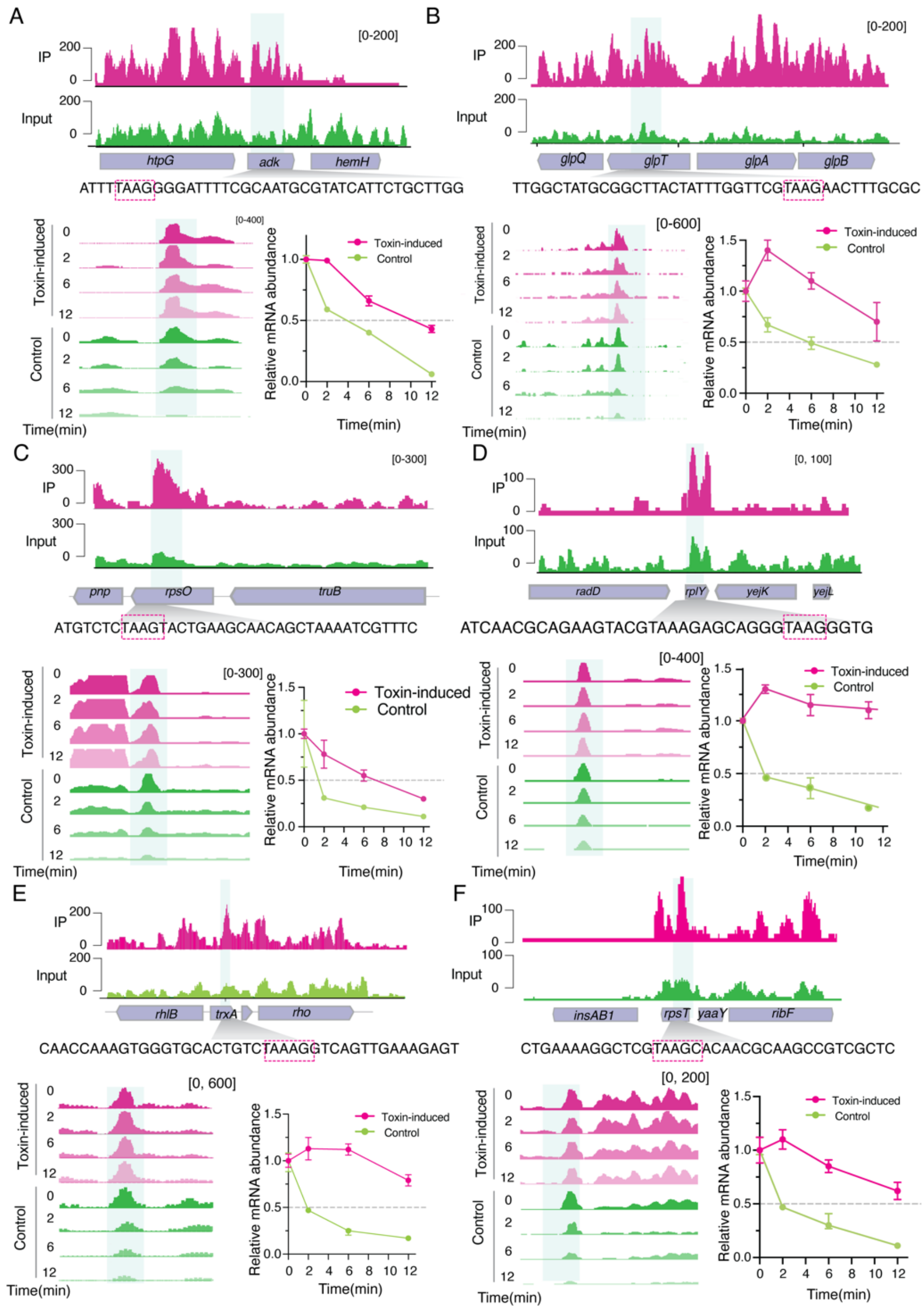

Figure S6. Toxin-dependent stabilization of additional RhsP2-associated mRNA targets.

(A–F) Genome-browser and rifampicin-chase validation of additional representative RhsP2-associated mRNA targets: *adk* (A), *glpT* (B), *rpsO* (C), *rplY* (D), *trxA* (E), and *rpsT* (F). For each locus, the upper tracks show RhsP2* eCLIP-seq IP and matched input signals. Gene models and UAAG-related motif positions are shown below. Lower panels show rifampicin-chase RNA-seq coverage (left) and qRT-PCR decay analyses (right) in control and RhsP2-induced cells.

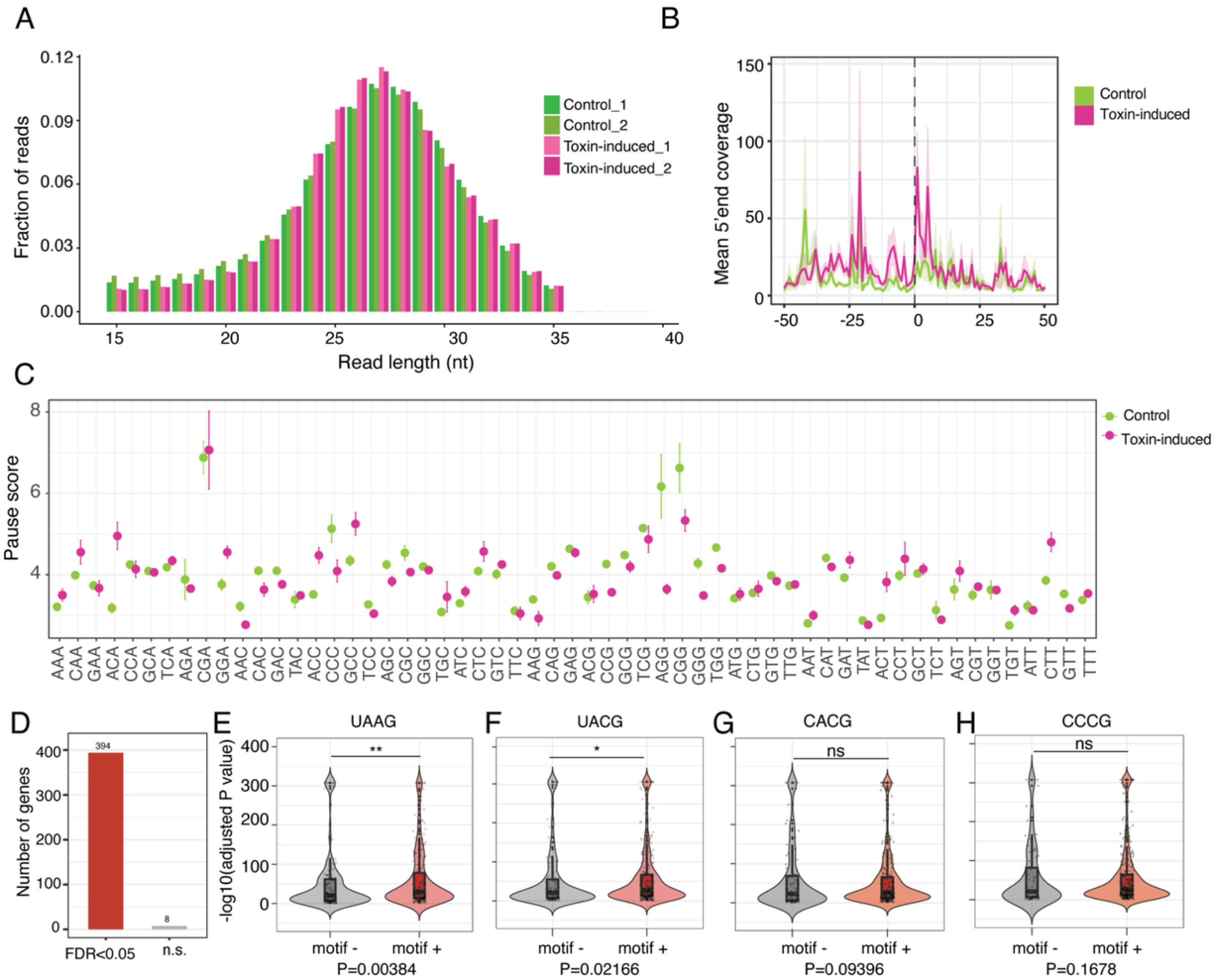

Figure S7. Transcriptome-wide analysis of RhsP2-dependent ribosome occupancy and pausing

(A) Read-length distribution of monosome ribo-seq libraries from control and RhsP2-induced samples. Ribosome-protected fragments (RPFs) are enriched within the expected monosome footprint size range, with most reads falling between approximately 26 and 30 nt.

(B) Metagene profile of RPF 5′-end coverage centered on motif-proximal ADP-ribosylation sites. RhsP2-induced samples show localized enrichment of RPF 5′ ends near the centered site relative to control samples.

(C) Codon-level pause scores for the remaining sense codons not highlighted in Figure 3F. Control and RhsP2-induced samples are shown to provide the global background distribution of codon-level pausing.

(D) Number of genes showing significant or non-significant RhsP2-dependent changes in ribosome P-site distribution. Among 402 genes analyzed, 394 showed significant changes after Benjamini–Hochberg correction.

(E–H) Distributions of $-\log_{10}$(adjusted P values) for genes containing motif (motif+) or lacking motif (motif−) (E) UAAG, (F) UACG, (G) CACG, or (H) CCCG motifs. Statistical comparisons between motif+ and motif− groups were determined using a Wilcoxon rank-sum test (* $P < 0.05$, ** $P < 0.01$, ns = not significant).

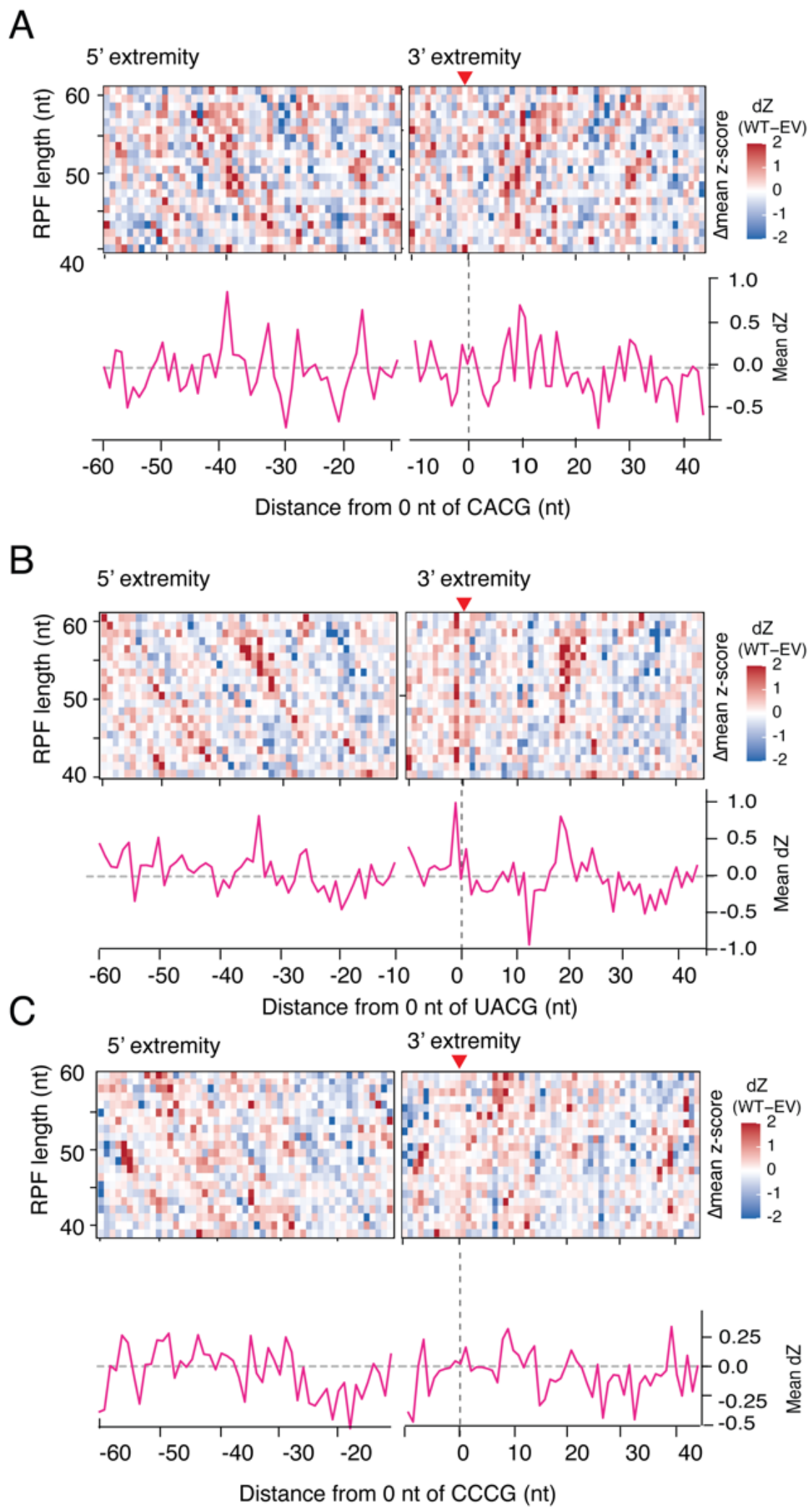

Figure S8. Motif-centered disome-seq analysis of RhsP2 target sequence variants. (A–C) Disome-seq metaprofiles aligned to the first nucleotide of (A) CACG, (B) UACG, or (C) CCCG sites, each defined as position 0. Heatmaps show differential Z-score signal (dZ = RhsP2-induced – control) for the 5′- and 3′-extremities of disome-protected fragments across disome-sized read lengths. Arrowheads indicate the anchor position used for alignment. Traces below each heatmap show the mean dZ signal averaged across read lengths at each relative position.

Table S1 Strains used in this study

Organism	Genotype	Description	References
E. coli BL21 (DE3) pLysS	F- ompT gal dcm lon hsdSB(rB$^-$ mB$^-$) λ (DE3) pLysS(cm^R)	Protein expression strain	Novagen
E. coli XL-1 Blue	*recA1 endA1 gyrA96 thi-1 hsdR17 supE44 relA1 lac* [F′ *proAB lacI^q* Z*ΔM15* Tn*10* (Tet^R)]	Cloning strain	Novagen

Table S2: Plasmids used in this study

Plasmid	Relevant features	Reference
pSCrhaB2-CV	Rhamnose-inducible expression vector carrying the P*rhaB* promoter, Tmp^R	Novagen
pSCrhaB2-CV::PA14_43100_1445-CT	Expression vector for $RhsP2_{tox}$	This study
pSCrhaB2-CV::PA14_43100_1445-CT_E1576Q-3×Flag	Expression vector for $RhsP2_{tox}^{E1576Q}$	This study
pSCrhaB2-CV::PA14_43100_1445-CT-3×Flag	Expression vector for $RhsP2_{tox}$3×Flag	This study
pPSV39-CV	Expression vector with lacI, lacUV5 promoter, C-terminal VSV-G tag, GmR	Novagen
pPSV39-CV::PA14_RS30650	Expression vector for *rhsI2*	(30)
pETDuet	Co-expression vector with lacI, T7 promoter, N-terminal His6 tag in MCS-1, Amp^R	Novagen
pETDuet::PA14_43100_1445-CT::PA14_RS30650	Co-expression vector of $RhsP2_{tox}$ and RhsI2	(26)
pETDuet::PA14_43100_1445-CT_E1576Q::PA14_RS30650	Co-expression vector of $RhsP2_{tox}^{E1576Q}$ and RhsI2	This study
pBAD33	Arabinose -inducible expression vector carrying the P*ara* promoter, Cmp^R	This study
pBAD33::mClover- stop	Cellular reporter expression	This study
pBAD33::mClover-3×Flag	Cellular reporter expression	This study
pBAD33::mClover-6×GUAAGA reporter-3×Flag	Cellular reporter expression	This study
pBAD33::T7-RBS-V5-mClover-stop	Dual-tag reporter template	This study
pBAD33::T7-RBS-V5-mClover-non-stalling-3×Flag	Dual-tag reporter template	This study
pBAD33::T7-RBS-V5-mClover-6×GUAAGA-3×Flag	Dual-tag reporter template	This study
pBAD33::T7-RBS-V5-mClover-SecM-3×Flag	Dual-tag reporter template	This study
pBAD33::tmRNA-smpB	Expression vector for tmRNA–SmpB	This study
pBAD33::arfA	Expression vector for ArfA	This study
pBAD33::arfB	Expression vector for ArfB	This study
pBAD33::tmRNA	Expression vector for tmRNA	This study

Table S3: Primers used in this study

Primer name	Sequence (5' to 3')	Description
16S F	TACCGCATAACGTCGCAAGA	qRT-PCR for 16S
16S R	CTGGACCGTGTCTCAGTTCC	rRNA
RT aptD F	GGTAATGAGCGTCTGGTGCT	qRT-PCR for aptD
RT aptD R	GAGTCGCTTTACCTACCGGG	
RT sodBF	CTACGGCAAGCACCATCAGA	qRT-PCR for sodB
RT sodBR	AAAGCTGCCAAAAGATGCGG	
RT appBF	CCGGCTTTTATGTGGCGATG	qRT-PCR for appB
RT appBR	ATTGCGGTGTGAAGGCAAAC	
RT aroBF	GGAACGTCTCGGGCAGTTTA	qRT-PCR for aroB
RT aroBR	GGACTTTCTTGTCACGCAGC	
RT rpsOF	GCAAACGACACCGGTTCTAC	qRT-PCR for rpsO
RT rpsOR	GCTCGATGAGCTGGGTGTAA	
RT pgkF	ACGTTCGCTTCAACAAAGGC	qRT-PCR for pgkF
RT pgkR	CCACCAACGATAGCCACCAT	
RT katGF	TTATCCTCGCGGGTAACGTG	qRT-PCR for katGF
RT katGR	ATCCAGATCCGGTTCCCAGA	
RT rpsTF	AAGCCGTCGCTCTATGATGC	qRT-PCR for rpsT
RT rpsTR	CACGATCGGTTGCATTTCGT	
RT adkF1	CAGACGCGATGAAAGAAGCG	qRT-PCR for adk
RT adkR1	CCGACGATACGGTCAACGAT	
RT cspEF	TCATTACTCCGGAAGACGGC	qRT-PCR for cspE
RT cspER	GAACTCTACGCGCTGACCTT	
RT trxAF	GGCGATCCTCGTCGATTTCT	qRT-PCR for trxA
RT trxAR	GCCCTGATATTCGTCAGCGA	
RT glpTF1	CGGCTTCCTGATCTACGGTC	qRT-PCR for glpT
RT glpTR1	GAAGTCCACGGTGTAGCCAA	
RT rplYF	TGACCATCGTTGTTGACGGT	qRT-PCR for rplY
RT rplYR	GAACGAAGTCGATGTGCTGC	
RT mcloverF	GAGCTGAAGGGCATCGACTT	qRT-PCR for
RT mcloverR	AACGTTGTGGCGGATCTTGA	mClover
RT motif Flag F	CCGCCGGGATTACACATGG	qRT-PCR for motif
RT motif Flag R	CACTTGTCATCGTCATCCTTGTAG	
RT tmRNA F	GCCTCGTAAAAAGCCGCAAA	qRT-PCR for
RT tmRNA R	TAAGAGCGGAGGCTAGGGAG	tmRNA
RT smpB F	TGGACTCATTGTACGGTCGC	qRT-PCR for smpB
RT smpB R	GCGACGCCGATTTTCACTTT	
RT arfA F	CCTTATTCCGACAGCGCGTA	qRT-PCR for arfA
RT arfA R	CCCCGATTGCCATGTTTTCC	

RT arfB F	GAGCTTGAGATCACCGCCAT	qRT-PCR for arfB
RT arfB R	GACAATCACGCCATCACTGC	
RT smrB F	TATGTTCGCCCGGATGTCAG	qRT-PCR for smrB
RT smrB R	AAAACACATGTTCACGGCGG	
pETDuet E1576Q up F	catcaccacagccaggatccgCTGGCCTCAAATAAAACTCGCTGT	For construction of pETDuet::RhsP2$_{tox}$^{E1576Q}::PA14_RS30650
pETDuet E1576Q up R	TGTTTCCAGCCCTCTCGGGCGG	
pETDuet E1576Q down F	GAGAGGGCTGGAAACAGAACAACGCACTATTCAAACT	
pETDuet E1576Q down R	cattatgcggccgcaagcttCTAGTCTATTACTTCGAAGTCTATTAGGTTCTTGTTGA	
pBAD smpB-tmRNA F	GTACCCGGGGATCCTCTAGAACGCTTatgACGAAGAAAAAAGCACA	For construction of pBAD33::tmRNA-smpB
pBAD smpB-tmRNA R	CCGCCAAAACAGCCAAGCTTTTAGGACTTCATCGGATGACTCTGGT	
pBAD arfA F	GTACCCGGGGATCCTCTAGAatgAGTCGATATCAGCATACTAAAGGGC	For construction of pBAD33::arfA
pBAD arfA R	CCGCCAAAACAGCCAAGCTTttaACAAGCGCCTGAAAGCAGAAG	
pBAD arfB F	GTACCCGGGGATCCTCTAGAatgATTGTGATTTCCCGACATGTTG	For construction of pBAD33::arfB
pBAD arfB R	CCGCCAAAACAGCCAAGCTTttaTTCCCGACCGCTGCG	
PBAD F tmRNA	GGTACCCGGGGATCCTCTAGAGGGGCTGATTCTGGATTCGAC	For construction of pBAD33::tmRNA
PBAD F tmRNA	ATCCGCCAAAACAGCCAAGCTTTGGTGGAGCTGGCGGG	
V5-mcl 1F	GCTCGGTACCCGGGGATCCTCTAGATAATACGACTCACTATAGGGAGGAGGT	For construction of T7-RBS-V5-mClover-stop
V5-mcl 1R	CGCCCTTGCTCACGCTGCCGCCGC	
V5-mcl 1_2F	CGGCAGCGTGAGCAAGGGCGAGGAGC	
V5-mcl 1_2R	CTCATCCGCCAAAACAGCCAAGCTTTCACTTGTACAGCTCGTCCATGC	
V5-mcl 2F	GCTCGGTACCCGGGGATCCTCTAGATAATACGACTCACTATAGGGAGGAGGT	For construction of pBAD33::T7-RBS-V5-mClover-non-stalling-3×Flag
V5-mcl 2R	CGCCCTTGCTCACGCTGCCGCCGC	
V5-mcl 2_2F	CGGCAGCGTGAGCAAGGGCGAGGAGC	
V5-mcl 2_2R	CTCATCCGCCAAAACAGCCAAGCTTTCACTTGTCATCGTCATCCTTGTAGTCGATGT	
V5-mcl 3F	GCTCGGTACCCGGGGATCCTCTAGATAATACGACTCACTATAGGGAGGAGGT	For construction of T7-RBS-V5-mClover-
V5-mcl 3R	CGCCCTTGCTCACGCTGCCGCCGC	

V5-mcl 1_3F	CGGCAGCGTGAGCAAGGGCGAGGAGC	6×GUAAGA-3×Flag
V5-mcl 1_3R	CTCATCCGCCAAAACAGCCAAGCTTTCACTTGTCATCGTCATCCTTGTAGTCGATGT	
V5-mcl 4F	GCTCGGTACCCGGGGATCCTCTAGATAATACGACTCACTATAGGGAGGAGGT	For construction of pBAD33::T7-RBS-V5-mClover-SecM-3×Flag
V5-mcl 4R	TATCAGGCTGAAAATCTTCTCTCATCCGCCAAAACAGCCAAGCTTTCACTTGTCATCGTCATCCTTGTAGTCGATGT	
